# Additive effect of Ruta graveolens bioactive phytoconstituents and cisplatin through PKC/MEK/ERK pathway in glioblastoma

**DOI:** 10.1016/j.omton.2026.201350

**Published:** 2026-10-01

**Authors:** Grazia Romano, Valentina Morgera, Antonio Pezone, Samantha Messina, Paolo Rosa, Manuela Iovinella, Claudia Ciniglia, Maria Teresa Gentile, Antonio Porcellini, Antonia Feola

## Main text

(Molecular Therapy: Oncology *34*, 1–14; September 2026)

In the originally published version of this article, the reference list contained incorrect references. The authors apologize for this mistake. The reference list has been corrected online.

The incorrect references are as follows:

33. Porcellini, A., Zuchegna, C., Casamassimi, A., de Cristofaro, T., Cuccurullo, J.P., Feliciello, A., Avvedimento, V.E., and Riccio, A. (2004). Ras inhibition amplifies cisplatin sensitivity of human glioblastoma. Biochem. Biophys. Res. Commun. *320*, 1148–1152. https://doi.org/10.1016/j.bbrc.2004.06.003.

36. Kwan, W., Shapiro, J.R., and Shapiro, W.R. (1982). Heterogeneous chemosensitivities of subpopulations of human glioma cells in culture. PubMed *42*, 992–998.

44. Rosa, P., Sforna, L., Carlomagno, S., Mangino, G., Miscusi, M., Prozzo, T., Baroncelli, L., Napolitano, F., Della Torre, A., Franciolini, F., et al. (2020). Overexpression of Large-Conductance Calcium-Activated Potassium Channels in Human Glioblastoma Stem-Like Cells and Their Role in Cell Migration. Int. J. Mol. Sci. *21*, 4101. https://doi.org/10.3390/ijms21114101.

45. Petridis, A.K., Salma, R., Anicic, M., Haase, A., Rolls, A., von Ammon, K., Steube, A., and Mühlenhoff, M. (2016). Polysialic Acid Sustains the Hypoxia-Induced Migration and Undifferentiated State of Human Glioblastoma Cells. Int. J. Mol. Sci. *17*, 1846. https://doi.org/10.3390/ijms17111846.

46. Scibetta, S., Pepe, G., Iuliano, M., Iaiza, A., Palazzo, E., Quadri, M., and Rosa, P. (2022). Polysialylation of Glioblastoma Cells Is Regulated by Autophagy Under Nutrient Deprivation. Cells, 7625. https://doi.org/10.3390/cells12010156.

47. Gattoni, G., Martín, J.R., Reyes, F., Sánchez-Porro, C., Feola, A., Zuchegna, C., Guerrero-Flores, S., Varcamonti, M., and Ricca, E. (2023). Genomic study and lipidomic bioassay of Leeuwenhoekiella parthenopeia. Front. Microbiol. *13*, 1090197. https://doi.org/10.3389/fmicb.2022.1090197.

The revised, correct references are as follows:

33. Messina, S., Leonetti, C., De Gregorio, G., Affatigato, V., Ragona, G., Frati, L., Zupi, G., Santoni, A., and Porcellini, A. (2004). Ras inhibition amplifies cisplatin sensitivity of human glioblastoma. Biochem. Biophys. Res. Commun. *320*, 493–500. https://doi.org/10.1016/j.bbrc.2004.06.003.

36. Yung, W.K., Shapiro, J.R., and Shapiro, W.R. (1982). Heterogeneous chemosensitivities of subpopulations of human glioma cells in culture. Cancer Res. *42*, 992–998.

44. Rosa, P., Sforna, L., Carlomagno, S., Mangino, G., Miscusi, M., Pessia, M., Franciolini, F., Calogero, A., and Catacuzzeno, L. (2017). Overexpression of Large-Conductance Calcium-Activated Potassium Channels in Human Glioblastoma Stem-Like Cells and Their Role in Cell Migration. J. Cell Physiol. *232*, 2478–2488. http://doi.org/10.1002/jcp.25592.

45. Rosa, P., Scibetta, S., Pepe, G., Mangino, G., Capocci, L., Moons, S.J., Boltje, T.J., Fazi, F., Petrozza, V., Di Pardo, A., et al. (2022). Polysialic Acid Sustains the Hypoxia-Induced Migration and Undifferentiated State of Human Glioblastoma Cells. Int. J. Mol. Sci. *23*, 9563. http://doi.org/10.3390/ijms23179563.

46. Scibetta, S., Pepe, G., Iuliano, M., Iaiza, A., Palazzo, E., Quadri, M., Boltje, T.J., Fazi, F., Petrozza, V., Di Bartolomeo, S., et al. (2025) Polysialylation of Glioblastoma Cells Is Regulated by Autophagy Under Nutrient Deprivation. Int. J. Mol. *26*, 7625. http://doi.org/10.3390/ijms26157625.

47. Gattoni, G., de la Haba, R.R., Martín, J., Reyes, F., Sánchez-Porro, C., Feola, A., Zuchegna, C., Guerrero-Flores, S., Varcamonti, M., Ricca, E., et al. (2023). Genomic study and lipidomic bioassay of Leeuwenhoekiella parthenopeia: A novel rare biosphere marine bacterium that inhibits tumor cell viability. Front. Microbiol. *13*, 1090197. http://doi.org/10.3389/fmicb.2022.1090197.

