# Supplementary material for: Additive effect of Ruta graveolens bioactive phytoconstituents and cisplatin through PKC/MEK/ERK pathway in glioblastoma
Source: Mol Ther Oncol. 2026 Aug 12;34(3):201320. doi: 10.1016/j.omton.2026.201320 (PMC13524684; doi:10.1016/j.omton.2026.201320)
Supplement: Supplementary file 1 [file mmc1.pdf]

OMTON, Volume *34*

Supplemental information

Additive effect of Ruta graveolens bioactive phytoconstituents and cisplatin through PKC/MEK/ERK pathway in glioblastoma

Grazia Romano, Valentina Morgera, Antonio Pezone, Samantha Messina, Paolo Rosa, Manuela Iovinella, Claudia Ciniglia, Maria Teresa Gentile, Antonio Porcellini, and Antonia Feola

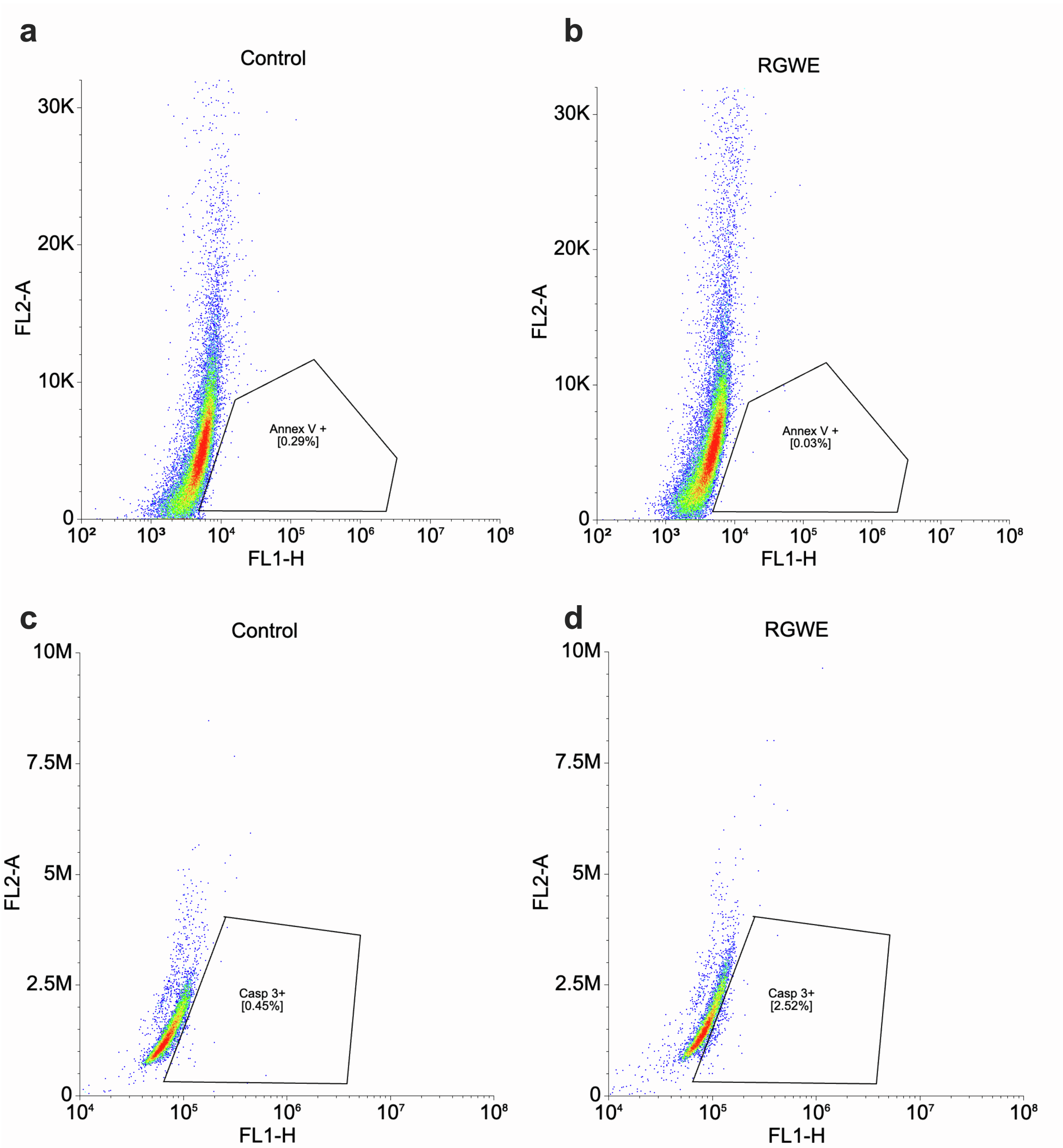

Figure S1: Apoptosis analysis via a cytofluorometric Annexin V-FITC/PI assay and a caspase-3 activation assay. U87MG cells were treated with 1 mg/ml RGWE for 48 h. The FL2-A/FL1-H dot plot represents the staining with AnnexV/PI (A-B), and Caspase3- active and PI (C-D) resulted in matching fractions of cells.

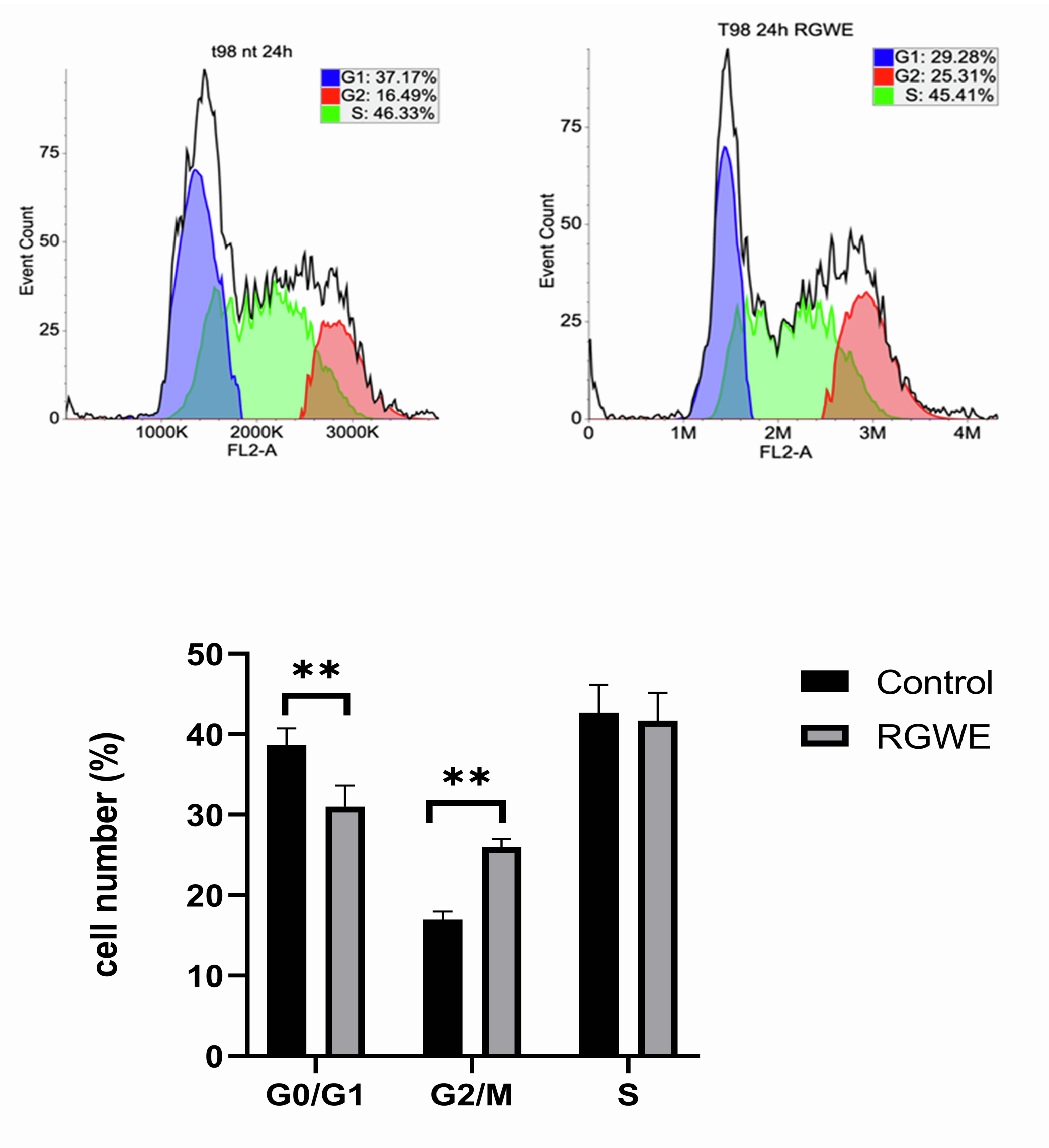

Figure S2. FACS flow cytometry analysis of the cell cycle in glioblastoma cell lines. (A) Representative flow cytometry cell cycle analysis of the T98G human glioblastoma cell line treated with 1 mg/ml RGWE for 24 h. The histogram shows the mean ± SD of the percentage of cells accumulated during treatment in each cell cycle phase of three independent experiments. The significance of the data obtained was tested via 2-way ANOVA with Sidak's multiple comparisons (**** $p < 0.0001$, ns: nonsignificant compared with untreated cells, control).

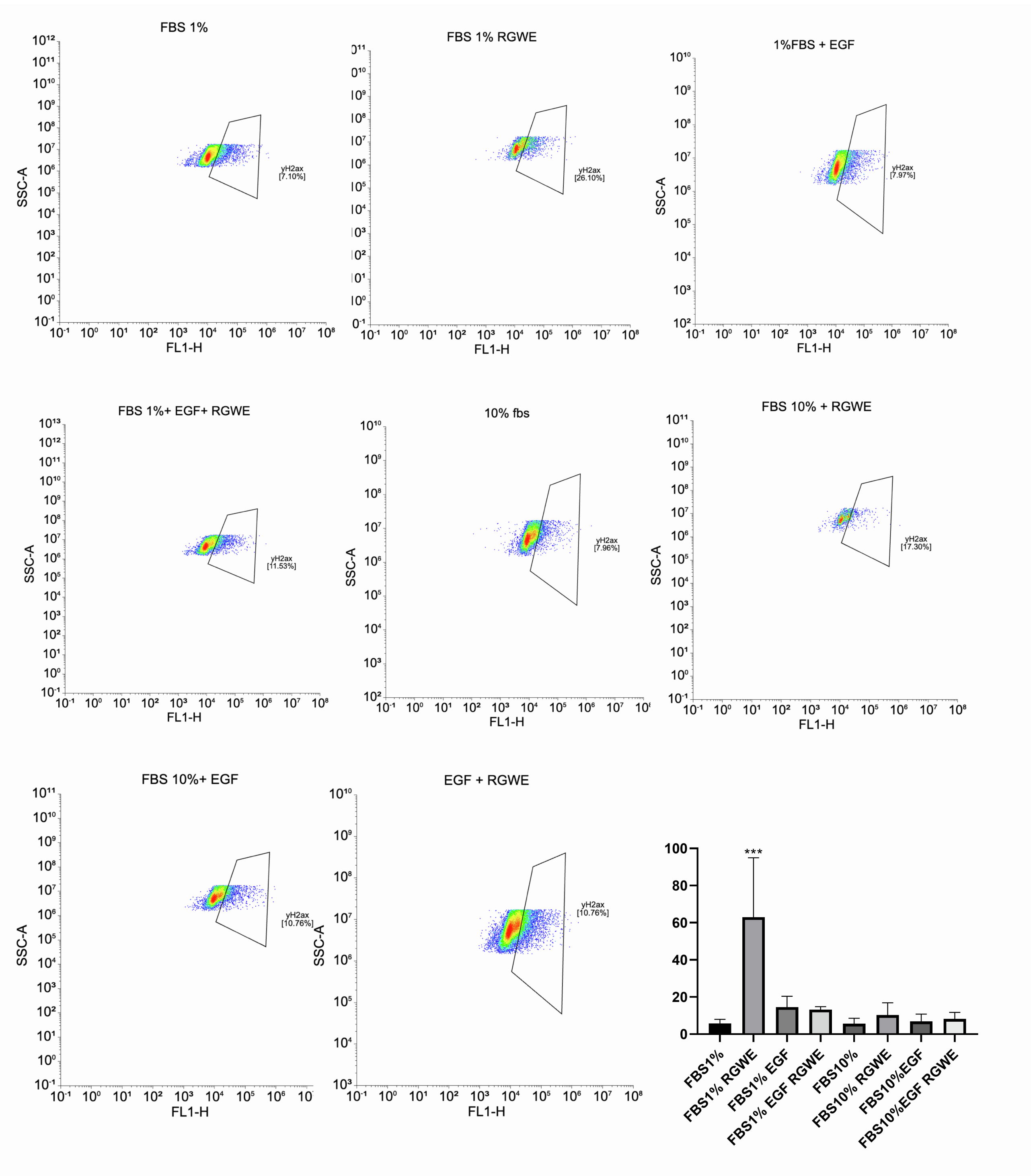

Figure S3. FACS flow cytometry analysis of γ-H2ax analysis in U-87 MG glioblastoma cell lines. (A) Representative flow cytometry Dot Plot of U-87 MG cells with 1 mg/ml RGWE for 24 h in the presence or in the absence of FBS1%, FBS 10% and EGF (5nM). The x-axis shows the intensity of fluorescence emitted by γ-H2ax antibody, while the y-axis shows cell size. The gating strategy is shown in the control not tested with the antibody. The histogram shows the mean ± SD of the percentage of cells γ-H2ax positive of three independent experiments. The significance of the data obtained was tested via 2-way ANOVA with Sidak's multiple comparisons (*** $p < 0.001$).

Table S1: List of DNA oligonucleotides used for real-time quantitative PCR (qPCR). The gene identification tag (ID) is shown on the left, the forward primer sequence (FW) and the reverse primer sequence (Rev) is on the right.

CCNE1	Fw	5' -GTGTCAAGTGGATGGTTCCATTTGCC- 3'
	Rv	5' -GCCCTATTTTGTTCAGACAACATGGC- 3'
TP53	Fw	5' -CTCAGCATCTTATCCGAGTGGAAGG- 3'
	Rv	5' -CACATGTAGTTGTAGTGGATGGTGG- 3'
P14	Fw	5' -CTCGTGCTGATGCTACTGAG- 3'
	Rv	5' -ACCAGCGTGTCCAGGAA- 3'
