# Supplementary material for: Additive effect of Ruta graveolens bioactive phytoconstituents and cisplatin through PKC/MEK/ERK pathway in glioblastoma
Source: Mol Ther Oncol. 2026 Aug 12;34(3):201320. doi: 10.1016/j.omton.2026.201320 (PMC13524684; doi:10.1016/j.omton.2026.201320)
Supplement: Supplementary file 2 [file mmc2.pdf]

Molecular Therapy
Oncology
Original Article

# Additive effect of Ruta graveolens bioactive phytoconstituents and cisplatin through PKC/MEK/ERK pathway in glioblastoma

Grazia Romano,[1] Valentina Morgera,[1] Antonio Pezone,[1] Samantha Messina,[2] Paolo Rosa,[3] Manuela Iovinella,[4] Claudia Ciniglia,[4] Maria Teresa Gentile,[4] Antonio Porcellini,[1,6] and Antonia Feola[1,5,6]

[1]Department of Biology, University of Naples "Federico II", Naples, Italy; [2]Department of Science, Roma Tre University, Rome, Italy; [3]Department of Medico-Surgical Sciences and Biotechnologies, Sapienza University of Rome, Polo Pontino, Latina, Italy; [4]Department of Environmental, Biological and Pharmaceutical Sciences and Technologies (DiSTABiF), University of Campania Luigi Vanvitelli, Caserta, Italy; [5]Department of Life Sciences, Health, and Health Professions, Link Campus University, Rome, Italy

**Glioblastoma multiforme (GBM) is a lethal malignancy with limited therapeutic options. Its aggressive progression and resistance to standard therapy necessitate the development of novel therapeutic strategies. Plant-based compounds have emerged as promising candidates for therapeutic development modulating key targets in cancer. The *Ruta graveolens* displays anti-inflammatory, analgesic, and antimicrobial properties. Herein, we report the anticancer potential of *R. graveolens* water extract (RGWE) on long-term cultures of human glioblastoma and elucidate the regulatory mechanisms driving its effect. Secondary stabilized cell cultures (U-87 MG, T98MG, and U-138 MG) and primary cell culture (FCN, MZC, and GL18-15) were either treated with RGWE and/or *cis*-diamminedichloroplatinum (cisplatin, CDDP). Cytostatic responses were assessed by cell viability (TB exclusion test), cell cycle (propidium iodide [PI] staining), and apoptosis (Annex-V and caspase-3) assays. We demonstrate that RGWE slows U-87 MG growth without affecting apoptosis and arrests the cell cycle in the G2/M phase. Mechanistically, RGWE interferes with PKC/MEK/ERK signaling pathway, as assessed by western blot analysis, through PKC inhibition in its active form. Furthermore, the combination of RGWE and cisplatin enables the use of a sublethal dose of the latter (0.2 μg/mL), thereby reducing cytotoxicity and the resistance to the drug. These findings reveal a novel mechanism by which RGWE controls glioblastoma growth, highlighting the therapeutic potential of targeting the PKC/MEK/ERK axis in glioma treatment.**

## INTRODUCTION

Glioblastoma multiforme (GBM) is one of the most aggressive and deadly forms of brain cancer and is characterized by a high ability to move and infiltrate healthy brain tissue, the ability to produce substances that promote the formation of new vessels, the difficulty of eradicating GBM through surgical approaches, and a poor prognosis.[1] Standard treatment approaches, including surgery, radiotherapy, and chemotherapy[2,3] offer limited efficacy because of early invasion of the brain parenchyma, therapy resistance, and the tumor microenvironment (TME).[4,5] Moreover, its complete surgical removal is almost impossible even after the introduction of temozolomide (TMZ, currently the standard cytotoxic drug for gliomas), and only 10% of patients survive beyond five years.[6] Since these approaches offer limited effectiveness, the need for novel therapeutic strategies has become urgent. Recent studies have highlighted the potential of novel approaches, such as combined targeted therapies, to improve outcomes.[7–9] Over the years, various natural products have gained attention because of their potential to modulate cancer pathways and enhance conventional therapies.[6,10,11] One such promising candidate is *Ruta graveolens*, commonly known as rue, a plant in the Rutaceae family that is traditionally used in folk medicine for its purported medicinal properties, including anti-inflammatory, analgesic, and antimicrobial effects.[12–14] Recent studies have begun to explore its potential role in cancer treatment, particularly because of its bioactive compounds. The water extract of *R. graveolens* could have potential applications in GBM therapy because several bioactive compounds found in the leaves may exhibit anticancer and neuroprotective properties.[13] In particular, *R. graveolens* extracts contain several phytochemicals, including flavonoids, such as rutin, quercetin, isorhamnetin, epigallocatechin,[6] and essential oils, which have demonstrated cytotoxic effects on various cancer cell lines. Some of these compounds are known to induce apoptosis in cancer cells, which is critical for combating the rapid and uncontrolled growth of tumor cells in lung cancer and melanoma.[15,16] The bioactive flavonoids in *R. graveolens* have shown anti-inflammatory effects, potentially reducing the inflammatory microenvironment that can

Received 16 December 2025; accepted 7 August 2026;
https://doi.org/10.1016/j.omton.2026.201320.

[6]These authors contributed equally

**Correspondence:** Antonia Feola, Department of Biology, University of Naples "Federico II", Naples, Italy.
**E-mail:** a.feola@unilink.it


© 2026 The Authors.
Published by Elsevier Inc. on behalf of The American Society of Gene and Cell Therapy.
This is an open access article under the CC BY license (http://creativecommons.org/licenses/by/4.0/).

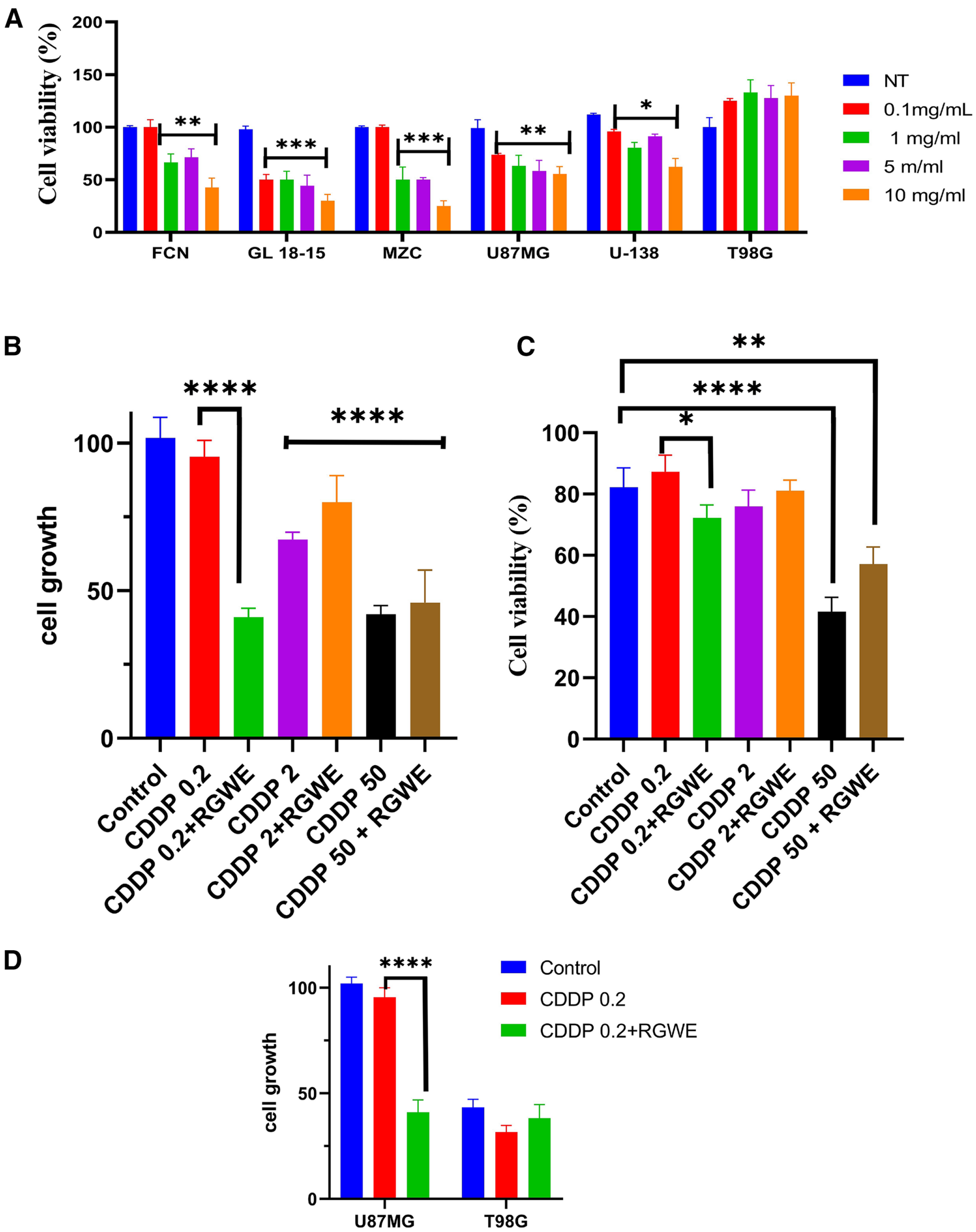


**Figure 1. RGWE in combination with CDDP impaired glioblastoma cell growth**

Trypan blue exclusion test on the U-87 MG U-138 MG, T98G, FCN, GL-18 15, and MZC cell lines. (A) Cells were treated with the RGWE concentrations as reported in figure. Untreated (NT) cells were used as positive controls. (B) U-87 MG cells were treated with 1 mg/mL RGWE and 0.2, 2, and 50 μg/mL CDDP as reported in figure. The untreated cells were used as control. (C) U-87 MG cells were treated as in B. (D) U-87 MG and T98G cells were treated with 1 mg/mL RGWE and 0.2 μg/mL CDDP as reported in figure. The error bars are representative of the SDs of three independent experiments performed in triplicate ($n \geq 9$). The significance of the data obtained was tested via one-way ANOVA with Sidak's multiple comparisons (****$p < 0.0001$, **$p < 0.001$, *$p < 0.05$ compared with untreated cells; ns: not significant).

exacerbate tumor growth.[14] Microenvironmental dependencies significantly modulate the behavior of cancer and stromal cells and regulate tumor progression, the chemotherapy response, and inflammatory cell infiltration.[17,18] The antioxidant properties of *R. graveolens* may help protect healthy brain cells from oxidative stress, a hallmark of GBM and other types of brain tumors. Water extracts may preserve normal cellular function and support the brain's response to treatment by scavenging free radicals and reducing oxidative damage. Some studies have indicated that *R. graveolens* extracts may have neuroprotective effects, which could be beneficial for patients with GBM, especially given the potential neurotoxicity of aggressive cancer treatments, such as chemotherapy and radiation.[13] Neuroprotection might reduce cognitive decline and other neurological side effects commonly associated with GBM therapy. Phytochemicals found in *R. graveolens* might enhance the effectiveness of conventional GBM therapies, such as chemotherapy or radiation. Some compounds have shown the ability to sensitize cancer cells to these treatments, increasing their susceptibility to cell death while protecting surrounding healthy tissue. While more research is needed to fully understand the specific mechanisms and therapeutic potential of *R. graveolens* in GBM, the combination of anticancer, anti-inflammatory, antioxidant, and neuroprotective properties suggest that its water extract could be a valuable supplement in GBM treatment. Further studies focusing on its mechanisms of action, preclinical evidence, and the underlying molecular pathways that its bioactive components may influence are necessary. Understanding the therapeutic potential of this plant extract could open new avenues for complementary or alternative treatments in the fight against one of the most challenging forms of brain cancer.

# RESULTS

## *R. graveolens* water extract impairs the viability of glioblastoma cell lines *in vitro*

The effect of *R. graveolens* water extract (RGWE) on cell viability was evaluated via a trypan blue exclusion test on secondary stabilized cell cultures (U-87 MG, T98MG, and U-138 MG) and primary cell culture (FCN, MZC, and GL18-15) treated with increasing concentrations of RGWE, ranging from 0.1 to 10 mg/mL, for 24 h. As shown in Figure 1A, RGWE induced a dose-dependent decrease in cell viability in all cell line tested but had no significant effect on T98G cells (Figure 1A). Specifically, GL18-15 and MZC primary cells showed a dose-dependent cell viability reduction after 24 h of RGWE stimulation with an efficacy higher than 55% between

1 and 5 mg/mL, whereas FCN cells needed RGWE 10 mg/mL to obtained similar results. The same test done on U-87 MG, T98MG, and U-138 MG secondary cell lines showed that to obtain the same effect (50% reduction in vitality) it was necessary to wait 48 h after treatment at the same doses in U-87 MG and in U-138 MG cells. This is conceivable if one considers that the secondary lines are more mutated than the primary ones. Notability, as reported before, RGWE did not show any effect on T98MG at all tested doses (Figure 1A). Based on these results, 1 mg/mL RGWE was selected for subsequent experiments. Afterward, we treated U-87 MG cells with increasing doses of CDDP, from 0.2 to 50 μg/mL, in the presence of 1 mg/mL RGWE for 48 h to test the possibility of complementary treatment. Our results demonstrated that, when we treated U-87 MG cells with a sublethal dose of CDDP (0.2 μg/mL; 0.7 μM)[19] in the presence of RGWE, cell growth significantly decreased compared with that in the presence of CDDP alone (Figure 1B), but the number of live cells did not appear to change significantly compared with that in the presence of CDDP alone (Figure 1C). Specifically, compared with RGWE treatment alone, RGWE cotreatment resulted in an overall 60% reduction in cell growth, with an additional 17% decrease in U-87 MG cell viability. Even in this case, the T98G cells retained their characteristic resistance (Figure 1D). These preliminary data suggest that RGWE could have an additive or synergistic effect with CDDP on the regulation of glioblastoma growth, allowing the use of a very low dose. To verify if the effects were cell dependent or contest dependent, we extended the experiments to a panel of both primary and secondary GBM cell lines, and we made a dose-dose matrix testing multiple RGWE and cisplatin concentrations. We have treated FCN, MZC, GL18-15, U-87 MG, and U-138 MG cells with increasing concentration of RGWE (from 0.1 to 10 mg/mL) and CDDP (from 0.2 to 50 μg/mL) in combination for 24 or 48 h if we did not see no effect before. Results showed in Figure 2 demonstrated an enhanced/additive (Bliss delta > +0.10 and $q \geq 0.05$) effect only at certain doses: for the U-87 MG cells we observed this effect only with 5 mg/mL RGWE with CDDP 2 μg/mL at 24 h, and with 1 mg/mL RGWE with CDDP 0.2 μg/mL at 48 h, as previously demonstrated; for MZC, we saw a synergism (Bliss delta > +0.10 and $q < 0.05$) at 1 mg/mL RGWE with CDDP 0.2 μg/mL, an enhanced/additive at 5 mg/mL RGWE in combination with CDDP 0.2 μg/mL and 1 mg/mL RGWE with CDDP 2 μg/mL only after 48 h of treatment. In contrast, the combination of two drugs on GL18-15, FCN, and U-138 MG cells did not show any synergic or enhanced/additive effects. At the end of this careful and detailed synergy analysis, what appears most evident to us it was that the almost of treatment combinations produced additive effects on cell viability of all cell lines tested, and this seemed dependent from the exposure time and the concentrations of each individual reagent used. This allowed us to identify a very low CDDP dose, lower than the lethal one (0.2 μg/mL) and short time expositions to obtain positive effects on glioblastoma cell viability reduction. It was also to be taken into consideration that there were other dose combinations for which the effect appears to be antagonistic. This makes the choice of doses of the two drugs and the exposure times even more important.

#### RGWE treatment affects cell cycle without interfering with apoptosis

To elucidate the impact of RGWE on cell growth, we further explored cell-cycle regulation via flow cytometry analysis. The results showed that RGWE induced significant accumulation of the glioblastoma cell lines U-87 MG and T98G in the G2/M phase of the cell cycle following 24 h of treatment; consequently, a reduction in the S phase was observed, whereas no significant effect on the G0/G1 phase of the cell cycle was detected (Figure 3A). These preliminary results suggest that RGWE could have an inhibitory effect on cell division by acting on the G2-M DNA damage checkpoint. Since cells with a defective G2-M checkpoint will undergo apoptosis or death after cell division, we tested the hypothesis that RGWE could impair cell viability, enhancing apoptosis. To this end, we performed a PI/annex V test and a caspase-3 active flow cytometry test, and both experiments demonstrated that RGWE did not increase the percentage of apoptotic dead cells after 48 h of treatment (Figure S1).

To verify the potency of RGWE in combination with CDDP, U-87 MG and T98G cells were treated with 1 mg/mL RGWE, 0.2 μg/mL CDDP or a combination of both, and cell growth was monitored over 48 h. As shown in Figure 2B, an additive effect was observed when RGWE was combined with cisplatin in U-87 MG cells, while T98G cells were resistant to both single treatments and their combination too. PI fluorescence-activated cell sorting (FACS) analysis confirmed a reduction in cell proliferation, especially under cotreatment conditions (Figure 3B). No significant variation was detected in T98G cells between CDDP alone and in the presence of RGWE, so we hypothesized that RGWE did not increase the lethality effect of CDDP on T98G cells (Figure 3B). Taken together, the data shown thus far suggest that RGWE might act on the inhibition of mitosis progression instead of the induction of apoptosis, and this effect together with the DNA damage caused by cisplatin, allows for more effective inhibition of cell division.

#### Involvement of MAPK signaling in the inhibitory activity of RGWE

The MEK-ERK pathway is essential, even if not the only pathway, for the mitogen-dependent induction of cyclins and transcription factors that allow the cell to pass the restriction point in late G1 phase and commit to S phase.[20] While the effect of RGWE on the MAPK transduction pathway is well understood,[6] the mechanism by which RGWE inhibits ERK1/2 phosphorylation remains unclear. For this purpose, U-87 MG cells were starved as described in the materials and methods section and then stimulated with 1 mg/mL RGWE for 10 min and 2 h under both starvation and growth conditions. This approach allowed us to assess the effects of RGWE under stress (1% fetal bovine serum [FBS]) and restored basal conditions (10% FBS). Following treatment, protein extracts were analyzed via western blotting. We decided to perform these experiments only on the U-87 MG cell line because T98G cells seem to be resistant to RGWE treatment. The results revealed a significant decrease in phospho-ERK (P-ERK) activation after 10 min, which persisted for up to 2 h under both starvation and growth conditions compared with that of the untreated cells under the same conditions (starvation and

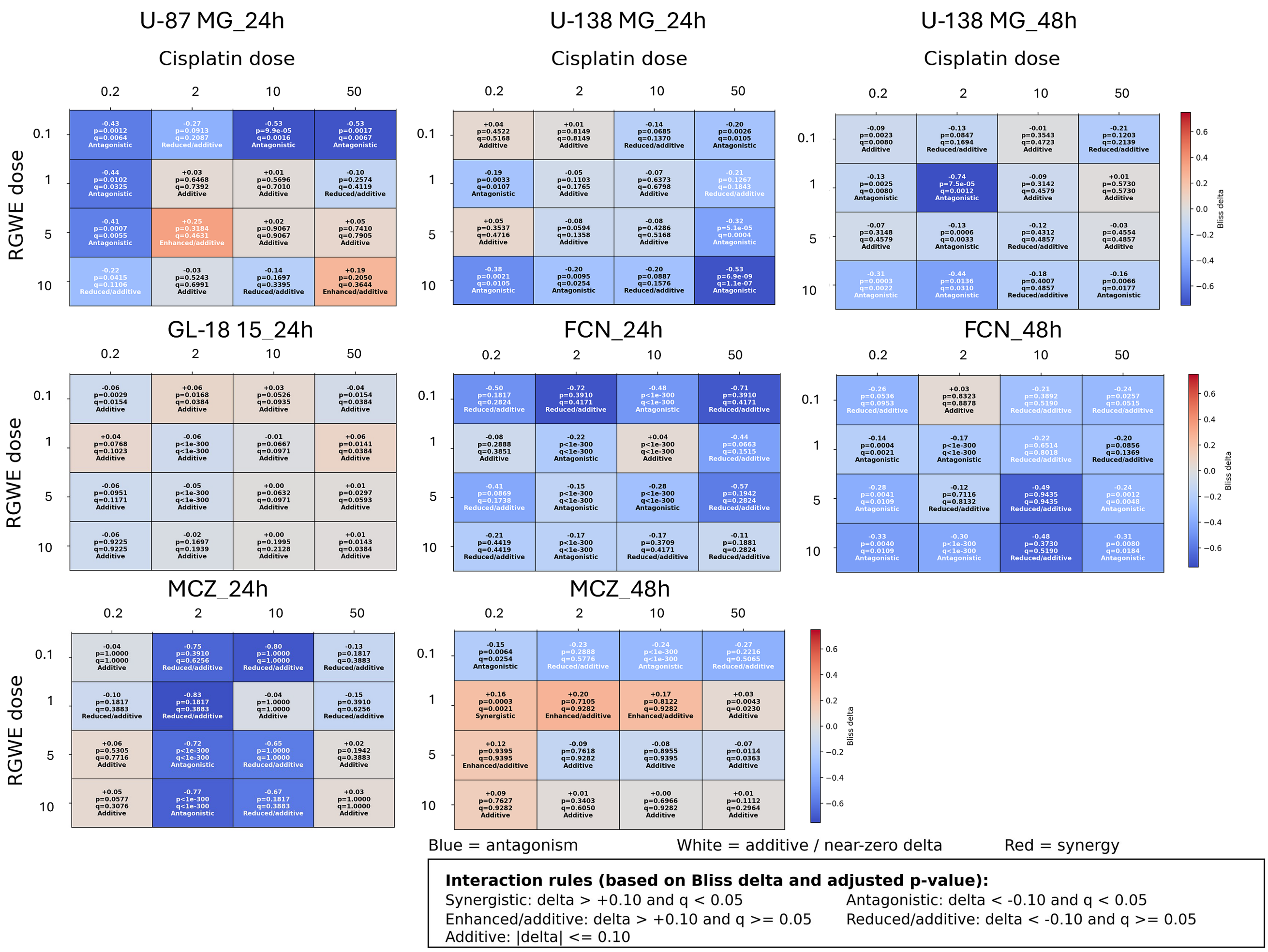


**Figure 2. Bliss independence RGWE and CDDP synergy analysis**

FCN, MZC, GL18-15, U-87 MG, and U-138 MG cell lines were treated with increasing concentration of RGWE (0.1–10 mg/mL) and CDDP (0.2–50 μg/mL) alone and in combination for 24 and 48 h. Cell viability was assessed by trypan blue exclusion test, and the results were converted to percent of cell viability. Positive Bliss-ΔE values indicate additive effect, consistent with a synergistic interaction, whereas negative Bliss-ΔE values indicate antagonism. Drug interactions were classified using both the magnitude of ΔE and the adjusted *p* value. Combinations with ΔE > 0.10 and adjusted *p* value <0.05 were classified as synergistic, whereas combinations with ΔE < −0.10 and adjusted *p* value <0.05 were classified as antagonistic. Non-significant positive or negative deviations beyond ±0.10 were reported as enhanced/additive or reduced/additive, respectively, while values within ±0.10 were considered additive. Statistical significance was indicated in the figure.

growth conditions) (Figure 4A). Notably, starvation induced an overall increase in P-ERK levels across all samples compared to control. These findings suggest that RGWE interferes with the MAPK signaling pathway by specifically inhibiting ERK phosphorylation, as evidenced by the marked reduction in P-ERK levels (Figure 4C). To clarify the role of RGWE in ERK1/2 signaling, U-87 MG cells were transfected with WT and active-Mek isoforms. After 2 h of starvation, the cells were treated with or without 1 mg/mL RGWE for 10 min or 2 h in the presence of 1% or 10% FBS. Western blot analysis confirmed that, compared with the WT isoform, the active-Mek isoform increased P-ERK levels under basal conditions. In active-Mek isoform-transfected U-87 MG cells, RGWE treatment significantly (****p* < 0.001) decreased ERK phosphorylation at 10 min but slightly increased ERK phosphorylation at 2 h under starvation conditions (1% FBS) (Figure 4B lines 1–5). In contrast, under restored basal conditions (10% FBS), RGWE was no longer capable of inhibiting ERK phosphorylation at either time point (Figure 4B lines 6–9). These findings support the hypothesis that RGWE inhibits the MEK/ERK1/2 signaling pathway through a negative upstream regulation of MEK. Since constitutive MEK activation prevents the previously observed decrease in ERK phosphorylation, we believe that RGWE could act on MEK activation in the context of the MAPK pathway.

### Dissecting the molecular mechanism of action of RGWE: The PKC/Mek/Erk signaling pathway

To identify the potential molecular mechanism of RGWE-dependent regulation upstream of MEK/ERK signaling, we looked for the major

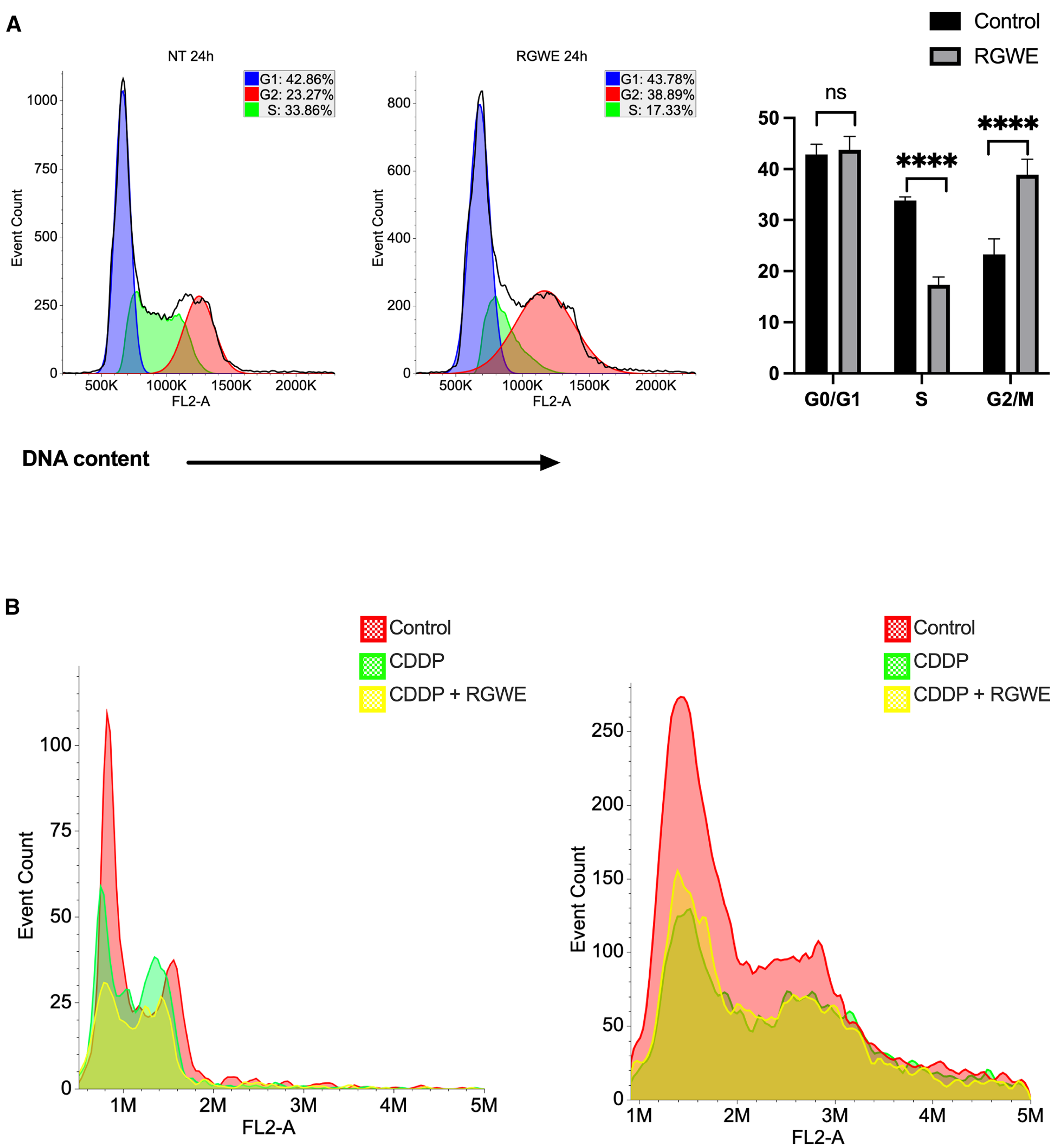


**Figure 3. FACS flow cytometry analysis of the cell cycle in glioblastoma cell lines**

(A) Representative flow cytometry cell cycle analysis of the U-87 MG human glioblastoma cell line treated with 1 mg/mL RGWE for 24 h. Histogram showing the mean ± SD of the percentage of cells that accumulated during each cell cycle phase in three independent experiments. The significance of the data obtained was tested via two-way ANOVA with Sidak's multiple comparisons (**** $p < 0.0001$, ns: nonsignificant compared with untreated cells, control). (B) Cells were treated with 0.2 μg/mL cisplatin in the presence or absence of 1 mg/mL RGWE. Untreated cells were used as controls. Histogram plots are representative flow cytometry cell-cycle analysis of U-87 MG (left) and T98G (right) human glioblastoma cell lines.

component in RGWE. LC-ESI-Q/TOF MS (liquid chromatography electrospray ionization quadrupole time-of-flight mass spectrometry) analysis[6] revealed that rutin was the major component of the extract but was present also quercetin 3-O-glucosyl-rhamnosyl-galactoside, isorhamnetin 3-O-rutinoside, isorhamnetin, and epigallocatechin 3-O-gallate. These flavonoids are well known to exert multiple pharmacological effects, including tumor suppression, carcinogen inactivation, cell-cycle arrest,[20] and apoptosis induction through their antioxidant and anti-inflammatory properties.[21–24] Moreover, recent evidence suggests that quercetin interacts with and permeates the lipid bilayer in a pH-dependent manner[25] and can influence $Ca^{2+}$ influx and metabolism. This, in turn, regulates the activation of

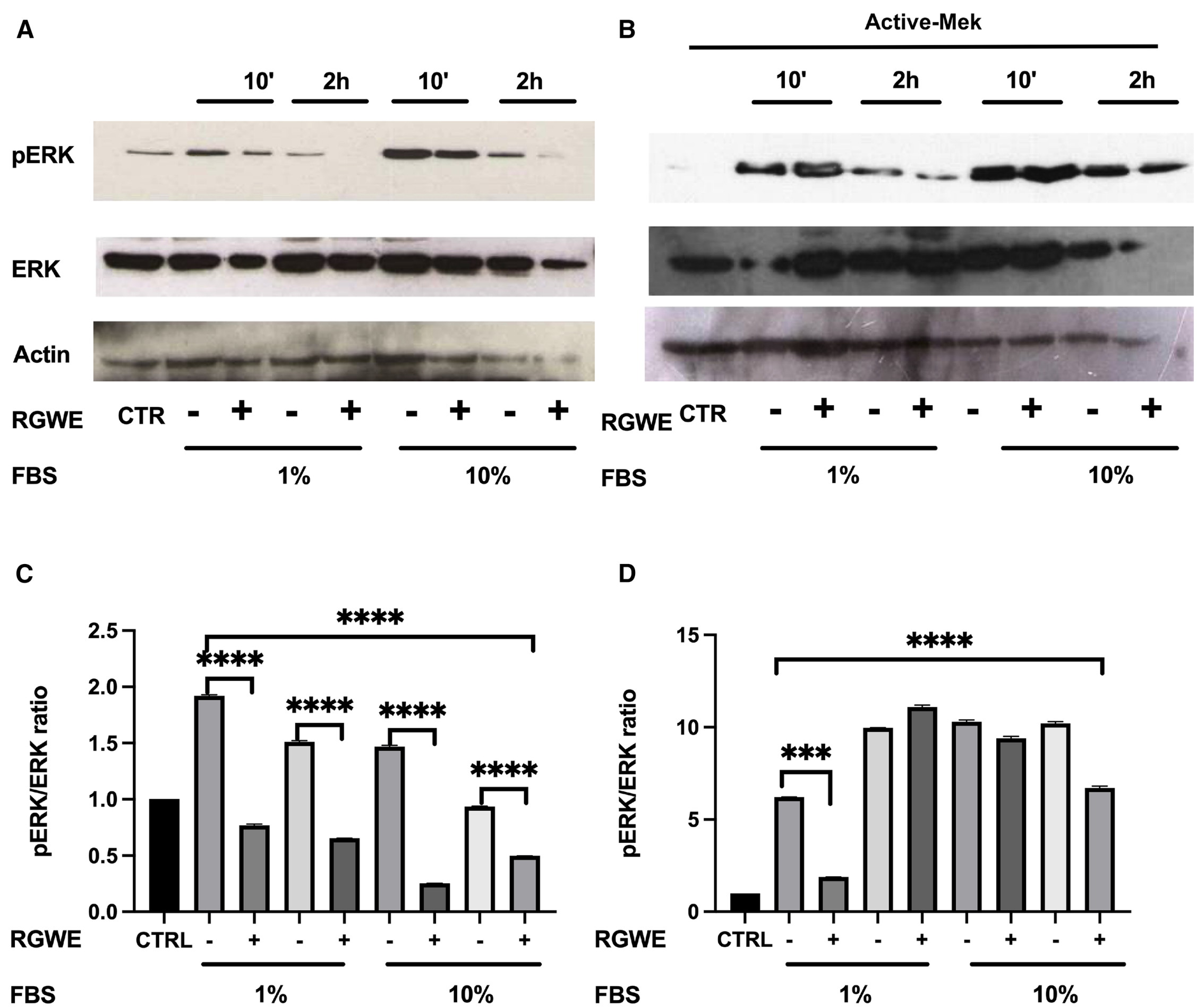


**Figure 4. RGWE inhibits MAPK signaling**

(A) Total protein extract obtained from U-87 MG cells stimulated with 1 mg/mL RGWE for 10′ and 2 h under starvation (1% FBS) and restored growth conditions (10% FBS) was analyzed via western blotting with anti-P-Erk, anti-Erk 1/2, and anti-actin antibodies. (B) U-87 MG cells transfected with the active-Mek isoform and treated with or without 1 mg/mL RGWE for 10 and 2 h in the presence of 1% and 10% FBS were analyzed via western blotting with anti-P-Erk, anti-Erk 1/2, and anti-actin antibodies. (C and D) The histogram represents the fold variation relative to the control P-Erk/Erk signal ratio obtained from different experiments. The cells that had not undergone starvation or subsequent treatments were used as controls. The values are expressed as the means ± SDs ($n = 7$). Differences between treatments were tested by two-way ANOVA with Dunnett's multiple comparisons (****$p < 0.0001$, ***$p < 0.001$, ns: nonsignificant compared with untreated cells, CTR).

protein kinase C (PKC) isozymes at the membrane.[26] Given that PKC activation can lead to the phosphorylation of several cellular proteins, including MAPK, we investigated whether RGWE could interfere with PKC-dependent signaling. To this end, U-87 MG cells were pretreated with 10 μM of PKC inhibitor (PKCi) for 30 min, followed by stimulation with 1 mg/mL RGWE for 10 min and 2 h. As expected, compared with starvation alone, PKCi treatment alone reduced P-ERK levels (Figure 5A line 2). Notably, subsequent RGWE administration significantly decreased P-ERK levels at 10 min, which persisted for up to 2 h. In contrast, under serum-induced reactivation of signaling (10% FBS), although P-ERK decreased at 10 min, this effect was no longer detectable at 2 h (Figures 5A–5C). These results suggested that RGWE acted upstream of PKC. To confirm this hypothesis, U-87 MG cells were transfected with an active-Mek mutant. Interestingly, the PKCi-induced inhibition of ERK phosphorylation was reversed within 10 min under all conditions. However, after 2 h, the signal appeared to decrease (Figures 5B–5D). When transfected cells (B) were compared with non-transfected cells (A), RGWE clearly increased the effect of the PKC inhibitor on ERK phosphorylation under serum-free conditions at both 10 min and 2 h. In contrast, under conditions of serum-induced phosphorylation reactivation, the inhibitory effect was lost, regardless of the presence of a PKC inhibitor. Finally, when cells overexpress the active-Mek mutant, not only does RGWE lose its inhibitory effect but the PKC inhibitor-mediated suppression of ERK phosphorylation is also significantly enhanced (Figure 5D). Taken together, these findings further support the hypothesis that RGWE modulates ERK phosphorylation upstream of

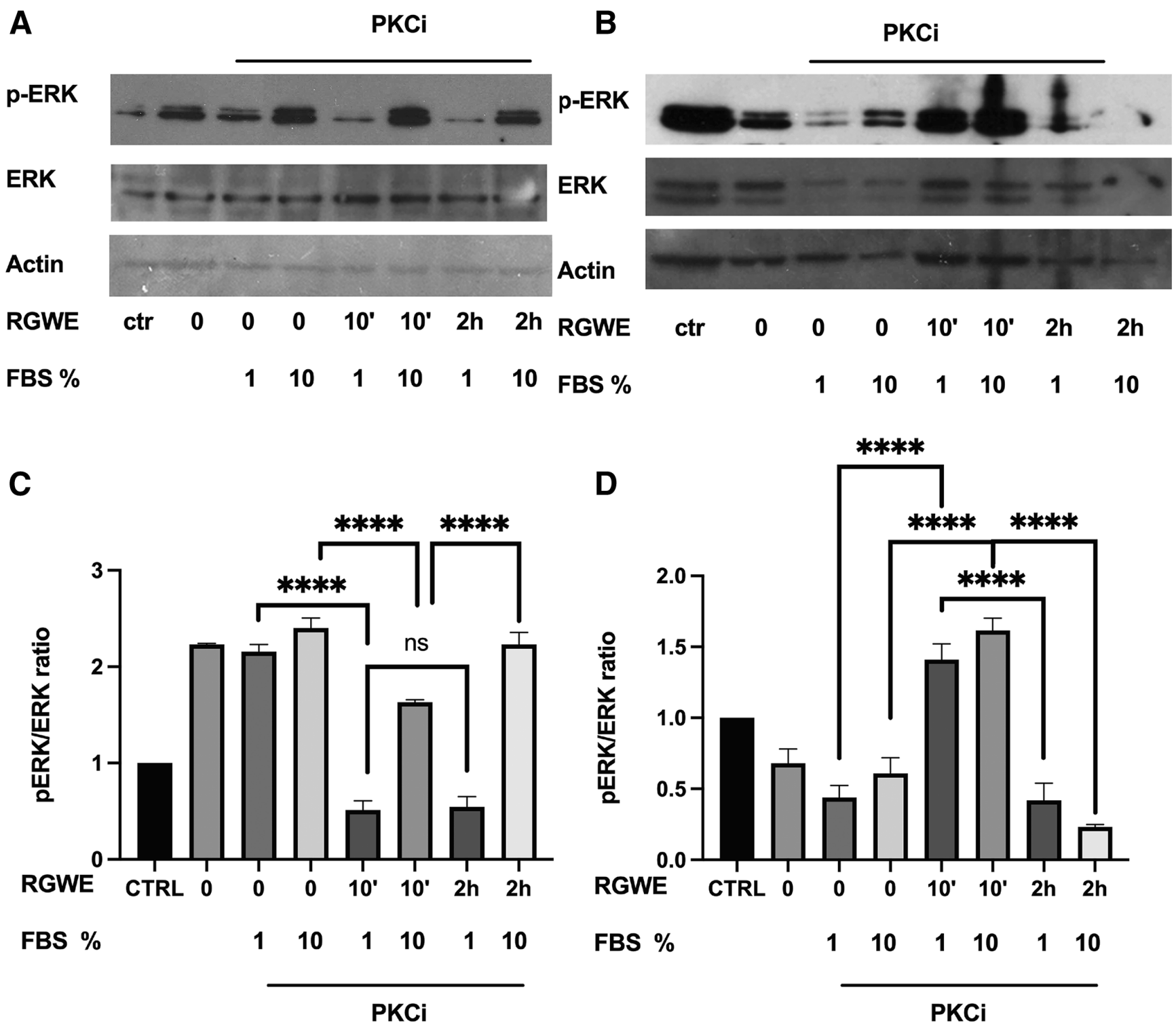


**Figure 5. RGWE acts through the PKC/Mek/Erk signaling pathway**

(A) Total protein extract obtained from U-87 MG cells was subjected to protein kinase C inhibitor (PKCi) for 30 min and then stimulated with 1 mg/mL RGWE for 10′ and 2 h in the presence of 1% or 10% FBS. (B) U-87 MG cells were previously transfected with the active-Mek isoform. Western blot analysis was performed with the indicated antibodies. (C and D) The histograms represent the mean ± SD ($n$ = 7) of P-Erk/Erk fold variation relative to the control (cells that had not undergone starvation or treatment). The values are expressed as differences between treatments and were tested for statistical significance via two-way ANOVA with Dunnett's multiple comparisons test (****$p < 0.0001$).

PKC and that its inhibitory activity is counteracted when MEK is constitutively active.

Moreover, to make us fully aware of the PCK-signaling implication in RGWE/MEK/ERK-dependent glioma growth inhibition, we treated glioblastoma cells with 1 μM PMA, a PKC activator (PKCa), for 30 min, stimulated them with 1 mg/mL RGWE for 48 h, and first, we assessed cell viability and cell cycle. Our results demonstrated that pretreatment with PKCa desensitized U-87 MG cells from the RGWE-dependent antiproliferative effect in terms of both viability and G2 phase cell-cycle arrest (Figures 6A–6C). Apparently, RGWE did not interfere with PKCa-dependent S-phase induction but attempted to enhance the percentage of cells that accumulated in the G0/G1 phase of the cell cycle. Finally, to investigate cell signaling, U-87 MG cells were pretreated with 1 μM PKCa for 30 min, and then RGWE was added for 10 min. As expected, PKCa alone increased P-ERK levels compared with those in the untreated condition (Figure 6D). At the same time, the addition of RGWE led to a significantly reduction in P-ERK levels within 10 min, an effect that was more pronounced than with RGWE alone and unexpected. So, in our opinion, these results support the involvement of the PKC/MEK/ERK pathway, especially if activated, as demonstrated by the decrease in P-PKC substrate levels when RGWE is added after PKCa treatment.

Finally, to demonstrate if there was a serum-dependent activity of RGWE, if this was limited to specific conditions or could benefit from specific context, we re-test key phenotypes under full serum and epidermal growth factor (EGF)-stimulated conditions to confirm robustness. Therefore, U-87 MG cells starved with 1% FBS for 2 h and then were treated with 1 mg/mL RGWE, in the presence and in the absence of EGF 5 nM or EGF alone for 24 h, then we analyzed the viability through trypan blue exclusion test, cell cycle by PI staining and FACS analysis. Results showed a statistically significant reduction in cell viability even in the presence of a single mitogenic stimulus (EGF). This reduction in viability correlates with an accumulation of cells in the G2/M phase of the cell cycle (Figures 7A and 7B), and with the ERK dephosphorylation RGWE-dependent even in the presence of EGF alone, although the EGF-mediated activation had been mild (Figure 7C). In addition, deeper cell-state analysis confirmed the RGWE cytostatic effects, because it induced a drastic increase in γH2AX (about 11-fold) in the first 24 h after nutrient deprivation, that it was not so pronounced following EGF stimulation. This could explain the accumulation in G2/M phase of the cell cycle observed. Then, to further explain the molecular mechanism through which RGWE controls cell cycle, we search for the expression of the key genes. qPCR data demonstrate that RGWE increased $p14^{CDKN2A}$ expression levels after 18 h of treatment both in the presence of FBS and in the presence of EGF. We found even an increase of TP53 expression levels at the same experimental time, which correlated with a decrease in CCNE1 expression levels after 1 h of RGWE treatment both in the presence of FBS and EGF, even if the last case the fold change is not significant (Figure 7D).

## DISCUSSION

A critical factor in the treatment of glioblastoma is the role of the blood-brain barrier (BBB), a highly selective physiological interface that restricts the penetration of most therapeutic agents into the central nervous system[27] In glioblastoma, however, the integrity of the BBB is often compromised due to abnormal angiogenesis and disrupted tight junctions, leading to increased permeability within tumor

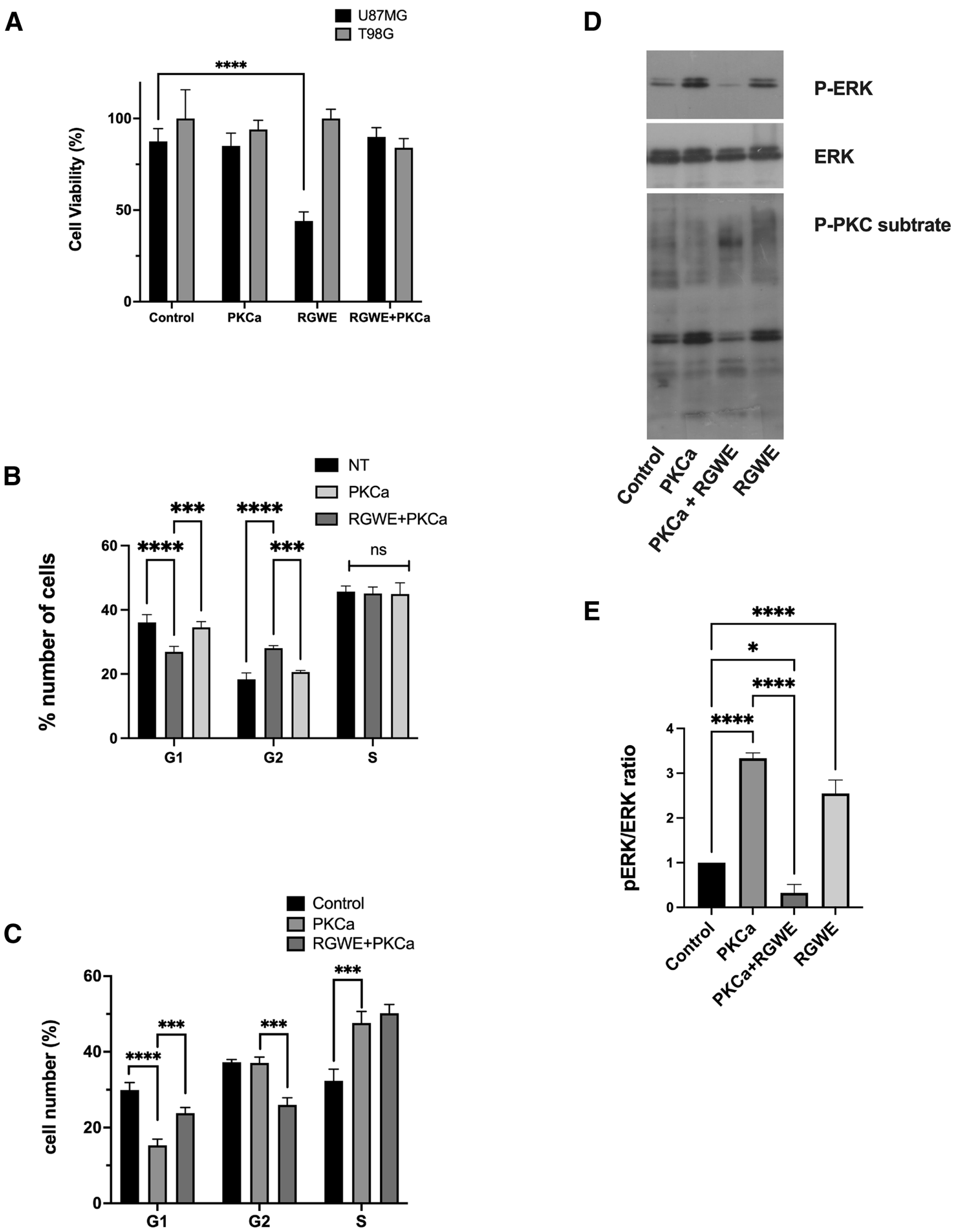


**Figure 6. RGWE affects cell growth through PKC-dependent signaling**

(A) Trypan blue exclusion test of U-87 MG and T98G cell lines pretreated with PKCa and then with RGWE alone or in combination for 24 h. Untreated (NT) cells were used as controls. The error bars are representative of the SDs of three independent experiments performed in triplicate. The significance of the data obtained was tested via one-way ANOVA with Sidak's multiple comparisons (****$p < 0.0001$ compared with untreated cells). (B) Histogram showing the mean ± SD of the percentage of cells that accumulated during each cell-cycle phase in three independent experiments. T98G cells were pretreated with PKCa and then with RGWE alone or in combination for 24 h. The significance of the data obtained was tested by two-way ANOVA with Sidak's multiple comparisons (****$p < 0.0001$ compared with untreated control cells). (C) U-87 MG cells treated as in B. The significance of the data obtained was tested via two-way ANOVA with Sidak's multiple comparisons (****$p < 0.0001$, ***$p < 0.001$ compared with the samples indicated in the figure). (D) Total protein extract obtained from U-87 MG cells stimulated with 1 mg/mL RGWE for 10′ in the presence or absence of PKCa was analyzed via western blotting with anti-P-Erk, anti-Erk 1/2, and anti-P-PKC substrate antibodies. (E) The histogram represents the fold variation relative to the control P-Erk/Erk signal ratio obtained from different experiments. The cells that had not undergone starvation or subsequent treatments were used as controls. The values are expressed as the means ± SDs ($n = 7$). Differences between treatments were tested by two-way ANOVA with Dunnett's multiple comparisons (****$p < 0.0001$, ***$p < 0.001$, ns: nonsignificant compared with untreated cells).

regions.[28] This phenomenon can facilitate the delivery of certain compounds that would otherwise have limited access to the brain. Some evidence suggests that certain polyphenols, such as resveratrol and quercetin, can be detected in brain tissue at low levels and may still exert biological effects.[29] Thus, the compromised BBB may further facilitate the access of RGWE polyphenols to the tumor core, but additional strategies such as formulation and delivery systems may be necessary to achieve therapeutically relevant concentrations throughout the tumor. This study revealed that the medicinal and culinary herb *R. graveolens* contains bioactive compounds that inhibit glioblastoma cell proliferation, reduce cell growth blocking cell-cycle progression in G2/M phase. Combination treatment with cisplatin has additive and dose reduction potential in glioblastoma cells, enabling the cisplatin dosage to be reduced to sublethal levels. Moreover, we identify the mechanism of action mediated by PKC/MEK/ERK signaling as possible target of the antiproliferative activity of RGWE. Our findings demonstrated that RGWE inhibits ERK1/2 phosphorylation through a mechanism that acts upstream of PKC and MEK cascade. The biochemical characterization of RGWE revealed a flavonoid-rich composition, with rutin as the major component, alongside other flavonoids such as quercetin derivatives and epigallocatechin gallate. These compounds are well documented for their antitumor properties,[30] including cell-cycle arrest, apoptosis induction, and modulation of intracellular signaling pathways. One key aspect of quercetin activity is its interaction with the plasma membrane, which influences $Ca^{2+}$ influx and metabolism. This, in turn, regulates PKC activation, a crucial event in cell signaling. In our experimental setting, PKC inhibition enables RGWE in lowering P-ERK levels exclusively under serum starvation conditions, which is probably due to the requirement of basal PKC activity. In the presence of serum, however, its effect on PKC activity was no longer evident, suggesting that external growth signals that strongly activate the pathway might override the inhibitory mechanism of RGWE. To further dissect the

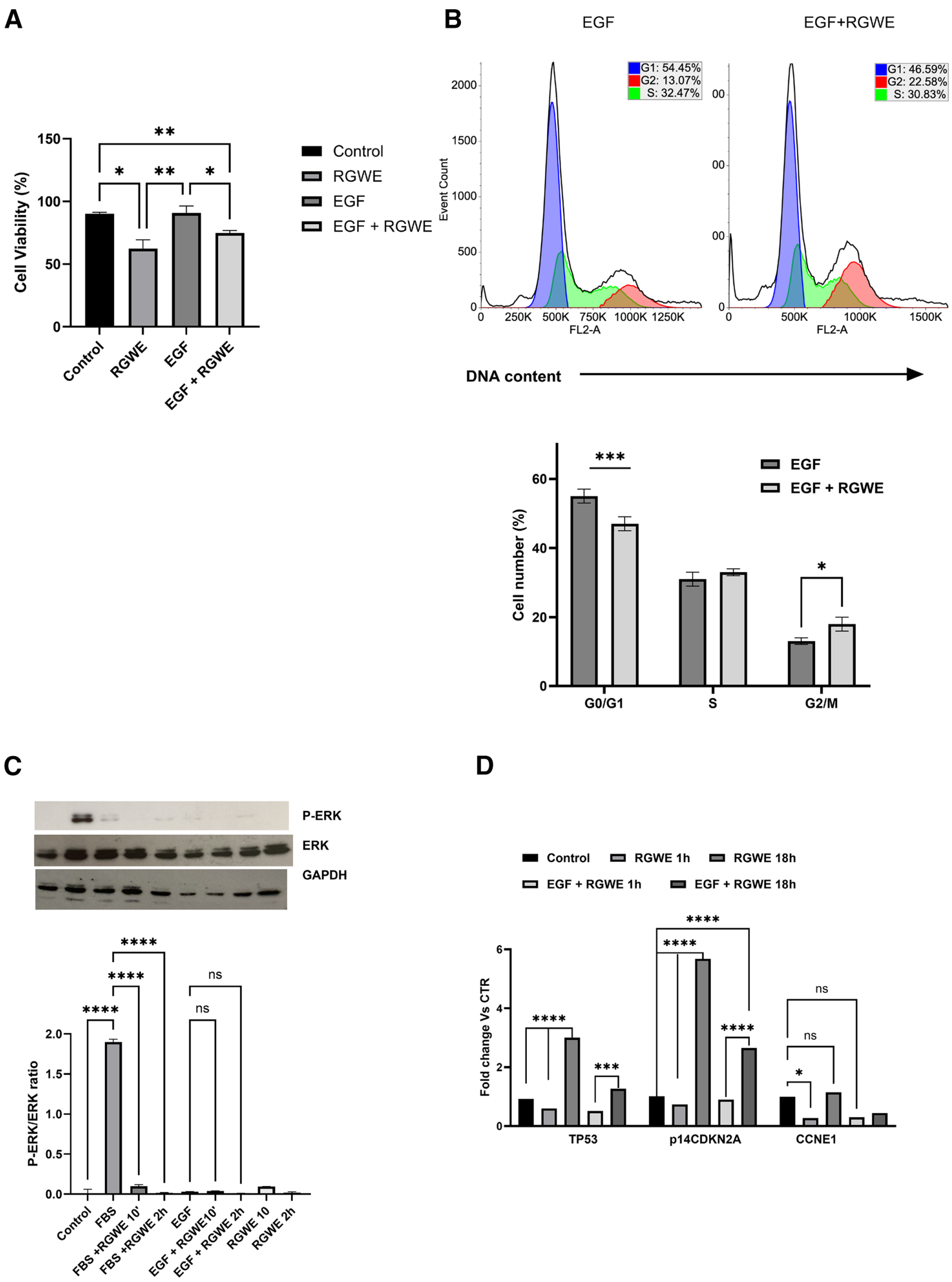


**Figure 7. RGWE acts on glioblastoma growth via PKC/ERK/MEC signaling in EGF-dependent microenviroment conditions**

(A) Trypan blue exclusion test of U-87 MG cell lines pretreated with RGWE alone or in combination with EGF for 24 h. Untreated (NT) cells were used as controls. The error bars are representative of the SDs of three independent experiments performed in triplicate. The significance of the data obtained was tested via one-way ANOVA with Sidak's multiple comparisons (****$p < 0.0001$ compared with untreated cells). (B) representative graphs of FACS cell-cycle analysis (upper part of the image), histogram showing the mean ± SD of the percentage of cells that accumulated during each cell cycle phase in three independent experiments (lower part of the image) in the same experimental conditions. (C) Total protein extract obtained from U-87 MG cells treated with 1 mg/mL RGWE for 10′ and 2 h in the presence of FBS 10% or EGF alone was analyzed via western blotting with the antibodies indicated in the figure. The histogram represents the fold variation relative to the control P-Erk/Erk signal ratio obtained from different experiments. Differences between treatments were tested by two-way ANOVA with Dunnett's multiple comparisons (****$p < 0.0001$, ***$p < 0.001$, ns: nonsignificant compared with untreated cells). (D) Total RNA extract obtained from U-87 MG cells treated with 1 mg/mL RGWE for 1 and 18 h in the presence and in the absence of EGF was analyzed via qPCR. The gene analyzed are reported in the figure. Differences between treatments were tested by two-way ANOVA with Dunnett's multiple comparisons (****$p < 0.0001$, ***$p < 0.001$, ns: nonsignificant compared with untreated cells). All values are expressed as the means ± SDs ($n = 7$).

mechanism of RGWE, we utilized an active-Mek mutant to bypass upstream regulatory controls. Interestingly, in cells overexpressing active-Mek, neither RGWE nor PKC inhibition suppressed ERK phosphorylation after 10 min. However, after 2 h, the signal appeared to diminish, suggesting possible feedback regulation or adaptation over time. Of note, in these transfected cells, the inhibitory effect of PKCi was even more pronounced, indicating potential cross-talk between PKC and MEK in regulating ERK activity. Conversely, we pretreated cells with 1 μM PKCa for 30 min and then added RGWE for 10 min. We confirmed that RGWE works through the PKC/MEK/ERK signaling pathway, especially if activated, since P-PKC substrate levels decrease when RGWE is added after PKCa treatment. Taken together, these data indicate that RGWE likely interferes with ERK activation at a step upstream of PKC and MEK. This finding is further supported by the observation that its effect is lost when MEK is constitutively active, meaning that RGWE does not directly inhibit MEK but rather modulates a preceding regulatory component. Given the known membrane-interacting properties of flavonoids such as quercetin, it is possible that RGWE influences signaling events at the plasma membrane level, potentially affecting the receptor-mediated activation of PKC or upstream kinases involved in ERK1/2 signaling. The RGWE activity on key phenomenon tested was maintain even under serum- or EGF-stimulated conditions, indicating that its effects are not limited to specific conditions but are robust across different proliferative contexts.

As confirmed in prostate, colon, and breast cancers,[31] RGWE induced significant accumulation of the glioblastoma cell lines U-87 MG and T98G in the G2/M phase of the cell cycle following 24 h of treatment. At the molecular level, RGWE induces the expression of key cell-cycle regulators, increasing p14$^{CDKN2A}$ and TP53 and reducing CCNE1, suggesting a role in controlling cell-cycle

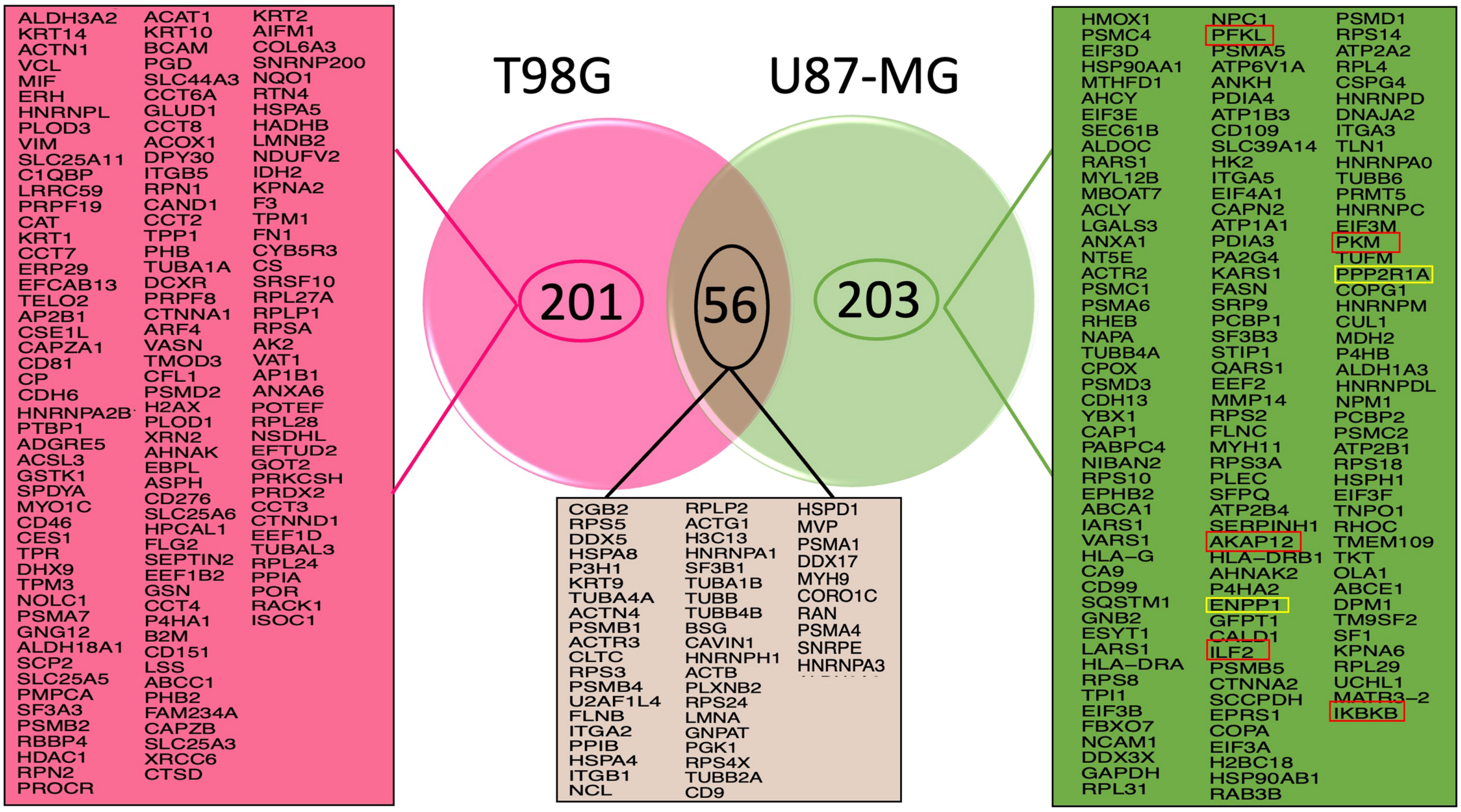


**Figure 8. Venn diagram of proteins identified in the U-87 MG and T98G cell lines**

Venn diagram of overlapping and unique proteins identified by means of mass spectrometry. The number of proteins in the overlapping and nonoverlapping areas is shown. Venn diagram proteins were grouped based on database annotation. Pink square reported the T98-G unique proteins, green square showed U-87 MG unique proteins, and beige square reported the common ones. In the red and yellow boxes are indicated protein kinases and protein phosphatases, respectively.

progression and in the antiproliferative response. Interestingly, we showed an additive effect between RGWE and cisplatin in all cell lines tested, but not in T98G cells that showed resistance to both single treatments and their combination. Our findings demonstrated that RGWE at different doses ranking from 0.1 to 5 mg/mL already after 24 h decreased the CDDP dose to a sublethal level (0.2 μg/mL; 0.7 μM), with an additive effect on the regulation of glioblastoma growth, both in term of cell viability and in cell-cycle progression regulation. Even the small doses tested (0.1 mg/mL) still reduced the viability of cells, suggesting the presence of long-acting components that inhibit cancer cell proliferation at low doses of the extract, illustrating the potential use of natural compounds as adjuvants or supplements to conventional therapeutic drugs.

Since combinatory therapy/treatment wants to come across the dependence from the exposure time and the concentrations of each individual reagent used, it was very important to identify the right dosage and timing, choosing the lowest dose and shortest time expositions to obtain positive effects on glioblastoma cell viability reduction. So, we propose RGWE as a new natural compound to inhibit MEK and interfere with glioblastoma proliferation in combination with a sublethal dose of CDDP, as demonstrated by the utilization of different glioblastoma cell lines. Although, we knew that TMZ is currently the standard-of-care treatment of glioblastoma, we choose CDDP based on our previous work on glioblastoma cells where we systematically reviewed experimental conditions, cell line types, drug exposure times, and IC50 values, which are dramatically different by 10- to 100-fold between cisplatin and TMZ.[20,32,33] Moreover, CDDP is the main chemotherapy used for multiple tumor types, and although it has been shown to have cytotoxic effects on human glioblastoma cells *in vitro*,[34,35] the response to clinical treatment is weak and does not improve the overall survival of patients with brain tumors. Within the area of combinatorial therapies, the use of MEK inhibitors in glioblastoma treatment has undergone increasing advances in clinical trials.[7,36–40]

Since previous studies reported that the water extract of *R. graveolens* induces cell death in several GBM cell lines, such as U138 and C6[6], the unexpected lack of effect on the T98G cell line prompted us to explore the potential differences between the two cell lines in terms of proteomics and, in particular, cell-surface proteomics because the water extract should not dissolve liposoluble molecules. In 2017, Gosh et al. published a cell surface membrane protein signature in glioblastoma[41] that allowed us to identify the differences between the two cell lines. As shown in the Venn diagrams, T98G and U-87 MG cells shared 56 membrane proteins, while 203 proteins were differentially expressed only in U-87 MG cells, which could be responsible for their different behavior (Figure 8). Among

these, 10 protein kinases and 2 protein phosphatases (see the red and yellow boxes, respectively) were selectively expressed in U-87 MG cells (green table). In particular, those that have attracted our attention include protein phosphatase 2, regulatory subunit alpha (PPP2R1A), which is the regulatory subunit of Ser/Thr phosphatases implicated in the negative control of cell growth and division, and the ectonucleotide pyrophosphatase/phosphodiesterase protein phosphatase 1 (E-NPP1), which is a transmembrane glycoprotein that cleaves a variety of substrates, including the phosphodiester bonds of nucleotides and nucleotide sugars. These findings lay the groundwork for further exploration of RGWE as a potential therapeutic agent targeting MAPK-driven oncogenic pathways. Future studies should focus on identifying the precise molecular targets of RGWE in the MAPK cascade. Investigating whether RGWE directly modulates receptor tyrosine kinase (RTK) activity, alters lipid raft composition, or interferes with protein phosphatases, such as PPP2R1A and E-NPP1 would provide valuable insights into its mechanism of action. Additionally, exploring its effects on other PKC isoforms and alternative ERK regulators may further elucidate its role in tumor signaling suppression.

In conclusion, since RGWE has been used for centuries as a spicing ingredient for food and to treat various illnesses, its extract contains candidate compounds that deserve to be further evaluated for their chemotherapeutic or adjuvant therapy potentials.

## MATERIALS AND METHODS

### Cell culture and plasmid transfection

Glioblastoma U-87 MG and T98-G cells were obtained from the American Type Culture Collection (ATCC, Rockville, MD) and cultured as previously described.[42] Primary glioblastoma MZC, FCN, and GL 18-15 were obtained and cultured as previously described.[43–46] The cells were transfected with purified plasmids via Superfect (QIAGEN) as previously reported.[47] The transfected cells were grown for 48 h in phenol red-free DMEM (Lonza; Verviers, Belgium), 10% (v/v) FBS (Biowest; Nuaillé, France), 1% pyruvate Na and 1% L-glutamine. Plasmids carrying human WT-MEK or its Ser217 and Ser221 residues in the mutated form of glutamic acid (reported as active-MEK) were obtained as previously described.[48] The pEGFP-C3 plasmid was obtained from BD Bioscience (#6082–1) and included to determine and normalize the transfection efficiency through FACS analyses. All the data used were derived from experiments with a transfection efficiency greater than 35%.

### RGWE preparation

RGWE was obtained from *R. graveolens*, a non-protected species. Leaves were collected from plants conserved at the Experimental Section of Medicinal Plants at the Botanical Garden of Naples, Italy. Whole leaves were harvested before the flowering stages, during spring, as previously described.[13] Technical variability was limited by a rigorous extraction procedure: 250 g of chopped leaves were boiled in 1 L of distilled water at 110°C for 60 min, followed by 0.22 μm filtration and lyophilization. Each extract batch underwent chemical characterization via RP-HPLC (reversed-phase high performance liquid chromatography) and LC ESI-Q/TOF mass spectrometry. This allowed for the consistent identification of a specific phytochemical "fingerprint" comprising five main flavonoid markers (i.e., rutin, quercetin 3-O-glucosyl-rhamnosyl-galactoside, isorhamnetin 3-O-rutinoside, isorhamnetin, epigallocatechin 3-O-gallate).[6] By monitoring the concentration of the major marker and the presence of the other identified flavonoids, we confirmed minimal batch-to-batch variability, ensuring that the biological effects observed are reproducible.

### Cellular treatments

For the cell signaling experiments, before each treatment, the cells were subjected to starvation with phenol red-free medium supplemented with 1% FBS for 2 h. RGWE was added at a final concentration of 1 mg/mL, unless otherwise specified, for 10 min and 2 h under both starvation conditions (1% FBS) and growth conditions (10% FBS). The cells were pretreated with 5 nM EGF (236-EG Sigma-Aldrich), 10 μM PKCi (PKC 20–28, 476480, Calbiochem) for 30 min or with 1 μM PKCa (P8139, Sigma-Aldrich) before the addition of 1 mg/mL RGWE for 10 min and/or 2 h. Cells that did not undergo any treatment were used as controls.

### Cell viability assay

Cell viability in response to treatment with cisplatin or RGWE alone or in combination was assessed via the trypan blue (Invitrogen) exclusion test. A total of $4 \times 10^4$ cells were seeded in 12-well plates and treated with RGWE at 1 mg/mL, 0.2, 2, or 50 μg/mL cisplatin in combination for 48 h. Then, the cells were detached, stained with a 0.4% (w/v) trypan blue solution, counted in a Burker chamber, and reported as the number of total cells, with the percentage of viable cells/total number being considered viable.

### Flow cytometry analysis

Cell-cycle analysis was performed via FACS as previously described.[49] Briefly, U-87 MG and T98-G cells were treated with 1 mg/mL RGWE, 0.2 μg/mL CDDP, or their combination for 48 h at 37°C. After two washes with PBS, the cells were fixed with 70% EtOH, permeabilized with 0.1% Triton and stained with 10 μg/mL propidium iodide (PI) with 5 μg/mL RNase in the dark at 4°C overnight. Apoptotic cells were detected via an annexin V-FITC/PI staining assay following the manufacturer's instructions as previously described[50] (A432; Leinco Technologies, Inc.) and via a FITC Active Caspase 3 Apoptosis Kit (550480 BD Bioscience), which selectively recognizes the active form of caspase-3 in human cells. Briefly, cells were treated with or without 1 mg/mL RGWE, incubated for 48 h and then stained following the manufacturer's recommendations. For γ-H2ax analysis, U-87 MG cells were starved or not as previously described and then treated with 1 mg/mL of RGWE in the presence and in the absence of 5 nM of EGF. After treatment cells were fixed 2 h with 70% of Et-OH, permeabilized for 10′ with Triton 0.1%, and then marked with anti-γ-H2ax antibody (BD #560443) following manufacture instructions. All cells were acquired with a threshold of FCS-H 80000 and gated in FSC-A/SSC-A based on their intrinsic

characteristics. When single cells in the flow cell pass through the BD Accuri-C6 laser beam, their FSC-A and FSC-H signals correlate linearly and plot along a relatively straight line, and single cells are gated on the diagonal by plotting FSC-A against FSC-H. At least 1,500 total events were acquired, of which 1,000 events were PI positive and analyzed. Fluorescence was determined by using an Accuri C6 flow cytometer (Becton Dickinson, Franklin Lakes, NJ, USA). All the data were analyzed with Floreada.io online software.

#### Immunoblotting analysis

U-87 MG cells, with or without MAPKK (MEK) or the mutant, were lysed with lysis buffer containing Tris-HCl (50 mM), EDTA (1 mM), Triton (1%), NaCl (150 mM), $MgCl_2$ (5 mM), EGTA (1 mM), 1× protease inhibitor, 1× PMSF, and 1× $Na_3VO_4$. The lysates were clarified by centrifugation at $12,000 \times g$ for 30 min at 4°C. The samples were separated by SDS−PAGE and subjected to western blotting. The nitrocellulose membranes were immunoblotted with antibodies against P-Erk (E-4) (sc-7383, Santa Cruz Biotechnology), Erk 1/2 (9102, Cell Signaling Technology), P-PKC-substrate (71652S, Cell Signaling Technology) and actin (F3777, Sigma−Aldrich); GAPDH (# 2275-PC-100, R&D Systems); at 1:1,000 in 3% BSA in T-TBS (0.1% Tween in TBS) overnight. After incubation with peroxidase-conjugated anti-mouse antibodies (1:5,000; Amersham-Biosciences), the proteins were visualized with an enhanced chemiluminescence (ECL) kit (Millipore). Band intensity was quantified with Fiji software.

#### RNA extraction and qPCR

Total RNA was extracted using TRIzol (Gibco/Invitrogen). cDNA was synthesized in 20 μL reaction containing 1 μg of total RNA, 100 U of Superscript III Reverse Transcriptase (Invitrogen) and 2 μL random hexamer (20 ng/μL) (Invitrogen). mRNA was reverse-transcribed for 1 h at 50°C, and the reaction was heat inactivated for 15 min at 70°C. The products were stored at −20°C. Quantitative reverse transcription polymerase chain reactions (PCRs) and quantitative PCRs (qPCRs) were performed on a 7500 Real-Time PCR System (Applied Biosystems) using the SYBR Green-detection system (FS Universal SYBR Green MasterRox/Roche Applied Science). The complete list of oligonucleotides used is reported in Table S1.

#### Bliss independence synergy analysis

The interaction between RGWE and cisplatin (CDDP) was quantitatively evaluated using the Bliss independence model. FCN, MZC, and GL18-15 primary glioblastoma cells, together with the established glioblastoma cell lines U-87 MG and U-138 MG, were treated for 24 and 48 h with increasing concentrations of RGWE (0.1–10 mg/mL) and CDDP (0.2–50 μg/mL), either alone or in combination. Cell death was assessed by trypan blue exclusion assay and expressed as the fraction or percentage of trypan blue-positive cells. The effects of RGWE and CDDP single treatments were first calculated and then used to estimate the expected additive effect for each drug combination according to the Bliss independence model. When effects were expressed as percentages, the Bliss-predicted additive effect was calculated as: EPRED = EA + EB − (EA × EB)/100, where EA and EB represent the effects induced by RGWE and CDDP alone, respectively. When effects were expressed as fractions ranging from 0 to 1, the equivalent formula was: EPRED = EA + EB − (EA × EB). The Bliss delta was calculated as: ΔE = EOBS – EPRED, where EOBS is the observed effect of the combination. Positive ΔE values indicate that the observed effect exceeds the Bliss-predicted additive effect, consistent with a synergistic interaction, whereas negative ΔE values indicate an effect lower than expected, consistent with antagonism. Drug interactions were classified using both the magnitude of ΔE and the adjusted $p$ value. Combinations with $\Delta E > 0.10$ and adjusted $p$ value $<0.05$ were classified as synergistic, whereas combinations with $\Delta E < -0.10$ and adjusted $p$ value $<0.05$ were classified as antagonistic. Non-significant positive or negative deviations beyond ±0.10 were reported as enhanced/additive or reduced/additive, respectively, while values within ±0.10 were considered additive. Statistical significance was indicated as follows: ns, not significant; *$p$ adj <0.05; **$p$ adj <0.01; ***$p$ adj <0.001; ****$p$ adj <0.0001.

#### Statistical analysis

All presented data are based on three independent experiments. The statistical analyses and graphs were prepared via Prism (8.0) (GraphPad Software). The bar plots in the graphs report the mean values (±SD). The tests of significant differences were analyzed via one-way and two-way ANOVA with Sidak's multiple comparison test and Dunnett's multiple comparison test.

#### Cell surface membrane protein analysis

Cell surface membrane protein data for T98G and U-87 MG cells were obtained from publicly available proteomic datasets.[41] Figure 7 displays the overlapping and unique cell surface membrane proteins identified by mass spectrometry.

## DATA AND CODE AVAILABILITY

All data relevant to the study are included in the article or uploaded as supplemental information and are available on reasonable request from the corresponding author.

## ACKNOWLEDGMENTS

This study was made possible by the care and abnegation of all participants despite the chronic difficulties afflicting the Italian scientific community. A. Porcellini acknowledges the support of the Italian Ministry of University (MUR) through the PRIN 2024 PNRR project (PNRR-MCNT2-2023-12377914).

## AUTHOR CONTRIBUTIONS

G.R. and V.M., contributed in methodology, validation, and investigation; A. Pezone performed the bioinformatic analysis; S.M. analyzed the results; M.I. and C.C., and M.T.G., contributed in conceptualization, writing – original draft, and preparation of figures; P.R., performed experiments on primary cells; A. Porcellini and A.F., supervised the experiments, analyzed the results, provided scientific interpretations, reviewed the original version. All authors have read and agreed to the published version of the manuscript.

## DECLARATION OF INTERESTS

The authors declare no competing interests.

## SUPPLEMENTAL INFORMATION

Supplemental information can be found online at https://doi.org/10.1016/j.omton.2026.201320.

## REFERENCES


1. Wu, W., Klockow, J.L., Zhang, M., Lafortune, F., Chang, E., Jin, L., Wu, Y., and Daldrup-Link, H.E. (2021). Glioblastoma multiforme (GBM): An overview of current therapies and mechanisms of resistance. Pharmacol. Res. *171*, 105780. https://doi.org/10.1016/j.phrs.2021.105780.

2. Bikfalvi, A., da Costa, C.A., Avril, T., Barnier, J.-V., Bauchet, L., Brisson, L., Cartron, P.F., Castel, H., Chevet, E., Chneiweiss, H., et al. (2023). Challenges in glioblastoma research: focus on the tumor microenvironment. Trends Cancer *9*, 9–27. https://doi.org/10.1016/j.trecan.2022.09.005.

3. van Tellingen, O., Yetkin-Arik, B., de Gooijer, M.C., Wesseling, P., Wurdinger, T., and de Vries, H.E. (2015). Overcoming the blood–brain tumor barrier for effective glioblastoma treatment. Drug Resist. Updat. *19*, 1–12. https://doi.org/10.1016/j.drup.2015.02.002.

4. Yang, F., Akhtar, M.N., Zhang, D., El-Mayta, R., Shin, J., Dorsey, J.F., Zhang, L., Xu, X., Guo, W., Bagley, S.J., et al. (2024). An immunosuppressive vascular niche drives macrophage polarization and immunotherapy resistance in glioblastoma. Sci. Adv. *10*, eadj4678. https://doi.org/10.1126/sciadv.adj4678.

5. Akiyama, T., Yasuda, T., Uchihara, T., Yasuda-Yoshihara, N., Tan, B.J.Y., Yonemura, A., Semba, T., Yamasaki, J., Komohara, Y., Ohnishi, K., et al. (2023). Stromal Reprogramming through Dual PDGFRα/β Blockade Boosts the Efficacy of Anti–PD-1 Immunotherapy in Fibrotic Tumors. Cancer Res. *83*, 753–770. https://doi.org/10.1158/0008-5472.can-22-1890.

6. Gentile, M.T., Russo, R., Pastorino, O., Cioffi, S., Barbieri, F., Illingworth, E.A., Grieco, M., Chambery, A., and Colucci-D'Amato, L. (2018). Ruta graveolens water extract inhibits cell-cell network formation in human umbilical endothelial cells via MEK-ERK1/2 pathway. Exp. Cell Res. *364*, 50–58. https://doi.org/10.1016/j.yexcr.2018.01.025.

7. Feola, A., Turitto, G., De Bellis, A., Alfieri, A., Bruscella, S., Meglio, V., Caiola, A., Migliore, F., Orabona, P., Porcellini, A., et al. (2025). Trametinib Combined with Pazopanib for Treating Malignant Peripheral Nerve Sheath Tumor in NF-1 Patients: A Case Report and a Review of the Literature. Annals of Case Reports *10*, 1–9. https://doi.org/10.29011/2574-7754.102156.

8. Jovčevska, I., Kočevar, N., and Komel, R. (2013). Glioma and glioblastoma – how much do we (not) know? Mol. Clin. Oncol. *1*, 935–941. https://doi.org/10.3892/mco.2013.172.

9. Zhai, K., Siddiqui, M., Abdellatif, B., Liskova, A., Kubatka, P., and Büsselberg, D. (2021). Natural Compounds in Glioblastoma Therapy: Preclinical Insights, Mechanistic Pathways, and Outlook. Cancers *13*, 2317. https://doi.org/10.3390/cancers13102317.

10. Angom, R.S., Nakka, N.M.R., and Bhattacharya, S. (2023). Advances in Glioblastoma Therapy: An Update on Current Approaches. Brain Sci. *13*, 1536. https://doi.org/10.3390/brainsci13111536.

11. Al Qaisi, Y., Alfarrayeh, I., Alsarayreh, A., Khleifat, K., and Abu-Nwas, N. (2024). Assessment of Antioxidant Potential, Cytotoxicity, and Anticancer Activity of Methanolic Extracts from Selected Wild Medicinal Plants. Phytomed. Plus *4*, 100534. https://doi.org/10.1016/j.phyplu.2024.100534.

12. Luo, P., Feng, X., Liu, S., and Jiang, Y. (2024). Traditional Uses, Phytochemistry, Pharmacology and Toxicology of Ruta graveolens L.: A Critical Review and Future Perspectives. Drug Des. Devel. Ther. *18*, 6459–6485. https://doi.org/10.2147/dddt.s494417.

13. Gentile, M.T., Ciniglia, C., Reccia, M.G., Volpicelli, F., Gatti, M., Thellung, S., Florio, T., Melone, M.A.B., and Colucci-D'Amato, L. (2015). Ruta graveolens L. Induces Death of Glioblastoma Cells and Neural Progenitors, but Not of Neurons, via ERK1/2 and AKT Activation. PLOS ONE *10*, e0118864. https://doi.org/10.1371/journal.pone.0118864.

14. Angeloni, C., Spencer, J.P.E., Leoncini, E., Biagi, P.L., and Hrelia, S. (2007). Role of quercetin and its in vivo metabolites in protecting H9c2 cells against oxidative stress. Biochimie *89*, 73–82. https://doi.org/10.1016/j.biochi.2006.09.006.

15. Duan, R., Liang, X., Chai, B., Zhou, Y., Du, H., Suo, Y., Chen, Z., Li, Q., and Huang, X. (2020). Isorhamnetin Induces Melanoma Cell Apoptosis via the PI3K/Akt and NF-κB Pathways. Biomed Res. Int. *2020*, 1057943. https://doi.org/10.1155/2020/1057943.

16. Lee, H.-J., Lee, H.-J., Lee, E.-O., Ko, S.-G., Bae, H.-S., Kim, C.-H., Ahn, K.-S., Lu, J., and Kim, S.-H. (2008). Mitochondria-cytochrome C-caspase-9 cascade mediates isorhamnetin-induced apoptosis. Cancer Lett. *270*, 342–353. https://doi.org/10.1016/j.canlet.2008.05.040.

17. Satari, A., Ghasemi, S., Habtemariam, S., Asgharian, S., and Lorigooini, Z. (2021). Rutin: A Flavonoid as an Effective Sensitizer for Anticancer Therapy; Insights into Multifaceted Mechanisms and Applicability for Combination Therapy. Evid. Based. Complement. Alternat. Med. *2021*, 9913179. https://doi.org/10.1155/2021/9913179.

18. Cruz, S.P., Zhang, Q., Devarajan, R., Paia, C., Luo, B., Zhang, K., Koivusalo, S., Qin, L., Xia, J., Ahtikoski, A., et al. (2024). Dampened Regulatory Circuitry of TEAD1/ITGA1/ITGA2 Promotes TGFβ1 Signaling to Orchestrate Prostate Cancer Progression. Adv. Sci. *11*, e2305547. https://doi.org/10.1002/advs.202305547.

19. Ghosh, D., Funk, C.C., Caballero, J., Shah, N., Rouleau, K., Earls, J.C., Soroceanu, L., Foltz, G., Cobbs, C.S., Price, N.D., et al. (2017). A Cell-Surface Membrane Protein Signature for Glioblastoma. Cell Syst. *4*, 516–529.e7. https://doi.org/10.1016/j.cels.2017.03.004.

20. Zuchegna, C., Leone, S., Romano, A., Porcellini, A., and Messina, S. (2024). KRAS is a molecular determinant of platinum responsiveness in glioblastoma. BMC Cancer *24*, 77. https://doi.org/10.1186/s12885-023-11758-6.

21. Villanueva, J., Yung, Y., Walker, J.L., and Assoian, R.K. (2007). ERK Activity and G1 Phase Progression: Identifying Dispensable Versus Essential Activities and Primary Versus Secondary Targets. Mol. Biol. Cell *18*, 1457–1463. https://doi.org/10.1091/mbc.e06-10-0908.

22. Pawlikowska-Pawlęga, B., Gruszecki, W.I., Misiak, L., Paduch, R., Piersiak, T., Zarzyka, B., Pawelec, J., and Gawron, A. (2007). Modification of membranes by quercetin, a naturally occurring flavonoid, via its incorporation in the polar head group. Biochim. Biophys. Acta *1768*, 2195–2204. https://doi.org/10.1016/j.bbamem.2007.05.027.

23. Jomova, K., Alomar, S.Y., Valko, R., Liska, J., Nepovimova, E., Kuca, K., and Valko, M. (2025). Flavonoids and Their Role in Oxidative stress, inflammation, and Human Diseases. Chem. Biol. Interact. *413*, 111489. https://doi.org/10.1016/j.cbi.2025.111489.

24. Chahar, M.K., Sharma, N., Dobhal, M.P., and Joshi, Y.C. (2011). Flavonoids: A versatile source of anticancer drugs. Pharmacogn. Rev. *5*, 1–12. https://doi.org/10.4103/0973-7847.79093.

25. Mir, S.A., Dar, A., Hamid, L., Nisar, N., Malik, J.A., Ali, T., and Bader, G.N. (2024). Flavonoids as promising molecules in the cancer therapy: An insight. Curr. Res. Pharmacol. Drug Discov. *6*, 100167. https://doi.org/10.1016/j.crphar.2023.100167.

26. Leite, N.B., Martins, D.B., Alvares, D.S., and Cabrera, M.P.D.S. (2022). Quercetin induces lipid domain-dependent permeability. Chem. Phys. Lipids *242*, 105160. https://doi.org/10.1016/j.chemphyslip.2021.105160.

27. Wu, D., Chen, Q., Chen, X., Han, F., Chen, Z., Wang, Y., and Wang, Y. (2023). The blood–brain barrier: structure, regulation, and drug delivery. Signal Transduct. Target. Ther. *8*, 217. https://doi.org/10.1038/s41392-023-01481-w.

28. Sica, F.S., Patrucco, M., Giambra, M., Ghizzi, M., Sesana, S., Vergani, B., Leone, B.E., Di Cristofori, A., Giussani, C.G., Nicolini, G., et al. (2025). Modulation of the blood–brain barrier permeability by patient-derived glioblastoma stem cell secretome. Sci. Rep. *15*, 37437. https://doi.org/10.1038/s41598-025-21272-3.

29. Vauzour, D. (2012). Dietary polyphenols as modulators of brain functions: biological actions and molecular mechanisms underpinning their beneficial effects. Oxid. Med. Cell. Longev. *2012*, 914273. https://doi.org/10.1155/2012/914273.

30. Fadlalla, K., Watson, A., Yehualaeshet, T., Turner, T., and Samuel, T. (2011). Ruta graveolens extract induces DNA damage pathways and blocks Akt activation to inhibit cancer cell proliferation and survival. Anticancer research *31*, 233–241.

31. Zhou, X., Yang, W., and Li, J. (2006). $Ca^{2+}$- and Protein Kinase C-dependent Signaling Pathway for Nuclear Factor-κB Activation, Inducible Nitric-oxide Synthase Expression, and Tumor Necrosis Factor-α Production in Lipopolysaccharide-stimulated Rat

Peritoneal Macrophages. J. Biol. Chem. *281*, 31337. https://doi.org/10.1074/jbc.m602739200.

32. Messina, S., Zuchegna, C., and Bruzzi, M. (2025). Chemotherapeutic nanoparticles for glioblastoma. Front. Oncol. *15*, 1641752. https://doi.org/10.3389/fonc.2025.1641752.

33. Porcellini, A., Zuchegna, C., Casamassimi, A., de Cristofaro, T., Cuccurullo, J.P., Feliciello, A., Avvedimento, V.E., and Riccio, A. (2004). Ras inhibition amplifies cisplatin sensitivity of human glioblastoma. Biochem. Biophys. Res. Commun. *320*, 1148–1152. https://doi.org/10.1016/j.bbrc.2004.06.003.

34. Singh, M., Verma, M., Pandey, S., Kumar, R., Khan, F., and Pandey, P. (2025). Anticancer Potential of Quercetin, Epigallocatechin Gallate, Kaempferol, Apigenin, and Curcumin against Several Human Carcinomas. Endocr. Metab. Immune Disord. Drug Targets *25*, 1148–1159. https://doi.org/10.2174/0118715303320523240910072723.

35. Iwata, K., Yung, W.K., and Fields, W.S. (1988). In Vitro Effects of ACNU, BCNU, CDDP, and Bleomycin on Human Glioma Cells. Neurol. Med. Chir. *28*, 965–969. https://doi.org/10.2176/nmc.28.965.

36. Kwan, W., Shapiro, J.R., and Shapiro, W.R. (1982). Heterogeneous chemosensitivities of subpopulations of human glioma cells in culture. PubMed *42*, 992–998.

37. Houweling, M., Abdul, U.K., Brahm, C., Lagerweij, T., Heukelom, S., Koken, P.W., Honeywell, R., Wedekind, L.E., Peters, G.J., Verheul, H., et al. (2023). Radio-sensitizing effect of MEK inhibition in glioblastoma in vitro and in vivo. J. Cancer Res. Clin. Oncol. *149*, 297–305. https://doi.org/10.1007/s00432-022-04483-3.

38. Schreck, K.C., Allen, A.N., Wang, J., and Pratilas, C.A. (2020). Combination MEK and mTOR inhibitor therapy is active in models of glioblastoma. Neurooncol. Adv. *2*, vdaa138. https://doi.org/10.1093/noajnl/vdaa138.

39. Singh, S., Dey, D., Barik, D., Mohapatra, I., Kim, S., Sharma, M., Prasad, S., Wang, P., Singh, A., and Singh, G. (2025). Glioblastoma at the crossroads: current understanding and future therapeutic horizons. Signal Transduct. Target. Ther. *10*, 213. https://doi.org/10.1038/s41392-025-02299-4.

40. Wagle, N., Sharma, A., Nguyen, M., Truong, J., Juarez, T.M., and Kesari, S. (2024). Neoadjuvant combination treatment with checkpoint inhibitors, chemotherapy, and BRAF/MEK inhibitors for BRAFV600E glioblastoma results in sustained response: A case report. Neurooncol. Adv. *6*, vdae110. https://doi.org/10.1093/noajnl/vdae110.

41. Boccellino, M., Galasso, G., Ambrosio, P., Stiuso, P., Lama, S., Di Zazzo, E., Schiavon, S., Vecchio, D., D'Ambrosio, L., Quagliuolo, L., et al. (2021). H9c2 Cardiomyocytes under Hypoxic Stress: Biological Effects Mediated by Sentinel Downstream Targets. Oxid. Med. Cell. Longev. *2021*, 6874146. https://doi.org/10.1155/2021/6874146.

42. Mao, X., Xu, J., Wang, W., Liang, C., Hua, J., Liu, J., Zhang, B., Meng, Q., Yu, X., and Shi, S. (2021). Crosstalk between cancer-associated fibroblasts and immune cells in the tumor microenvironment: new findings and future perspectives. Mol. Cancer *20*, 131. https://doi.org/10.1186/s12943-021-01428-1.

43. Calogero, A., Arcella, A., De Gregorio, G., Porcellini, A., Mercola, D., Liu, C., Lombari, V., Zani, M., Giannini, G., Gagliardi, F.M., et al. (2001). The early growth response gene EGR-1 behaves as a suppressor gene that is down-regulated independent of ARF/Mdm2 but not p53 alterations in fresh human gliomas. Clin. Cancer Res. *7*, 2788–2796. https://pubmed.ncbi.nlm.nih.gov/11555594/.

44. Rosa, P., Sforna, L., Carlomagno, S., Mangino, G., Miscusi, M., Prozzo, T., Baroncelli, L., Napolitano, F., Della Torre, A., Franciolini, F., et al. (2020). Overexpression of Large-Conductance Calcium-Activated Potassium Channels in Human Glioblastoma Stem-Like Cells and Their Role in Cell Migration. Int. J. Mol. Sci. *21*, 4101. https://doi.org/10.3390/ijms21114101.

45. Petridis, A.K., Salma, R., Anicic, M., Haase, A., Rolls, A., von Ammon, K., Steube, A., and Mühlenhoff, M. (2016). Polysialic Acid Sustains the Hypoxia-Induced Migration and Undifferentiated State of Human Glioblastoma Cells. Int. J. Mol. Sci. *17*, 1846. https://doi.org/10.3390/ijms17111846.

46. Scibetta, S., Pepe, G., Iuliano, M., Iaiza, A., Palazzo, E., Quadri, M., and Rosa, P. (2022). Polysialylation of Glioblastoma Cells Is Regulated by Autophagy Under Nutrient Deprivation. Cells, 7625. https://doi.org/10.3390/cells12010156.

47. Gattoni, G., Martín, J.R., Reyes, F., Sánchez-Porro, C., Feola, A., Zuchegna, C., Guerrero-Flores, S., Varcamonti, M., and Ricca, E. (2023). Genomic study and lipidomic bioassay of Leeuwenhoekiella parthenopeia. Front. Microbiol. *13*, 1090197. https://doi.org/10.3389/fmicb.2022.1090197.

48. Feola, A., Cimini, A., Migliucci, F., Iorio, R., Zuchegna, C., Rothenberger, R., Cito, L., Porcellini, A., Unteregger, G., Tombolini, V., et al. (2013). The inhibition of p85αPI3KSer83 phosphorylation prevents cell proliferation and invasion in prostate cancer cells. J. Cell. Biochem. *114*, 2114–2119. https://doi.org/10.1002/jcb.24558.

49. Cowley, S., Paterson, H., Kemp, P., and Marshall, C.J. (1994). Activation of MAP kinase kinase is necessary and sufficient for PC12 differentiation and for transformation of NIH 3T3 cells. Cell *77*, 841–852. https://doi.org/10.1016/0092-8674(94)90133-3.

50. Di Zazzo, E., Feola, A., Zuchegna, C., Romanos, A., Donini, C.F., Bartollino, S., Costagliola, C., Frunzio, R., Laccetti, P., Di Domenico, M., et al. (2014). The p85 Regulatory Subunit of PI3K Mediates cAMP-PKA and Insulin Biological Effects on MCF-7 Cell Growth and Motility. ScientificWorldJournal. *2014*, 565839. https://doi.org/10.1155/2014/565839.

**OMTON, Volume *34***

## Supplemental information

# Additive effect of Ruta graveolens bioactive phytoconstituents and cisplatin through PKC/MEK/ERK pathway in glioblastoma

**Grazia Romano, Valentina Morgera, Antonio Pezone, Samantha Messina, Paolo Rosa, Manuela Iovinella, Claudia Ciniglia, Maria Teresa Gentile, Antonio Porcellini, and Antonia Feola**

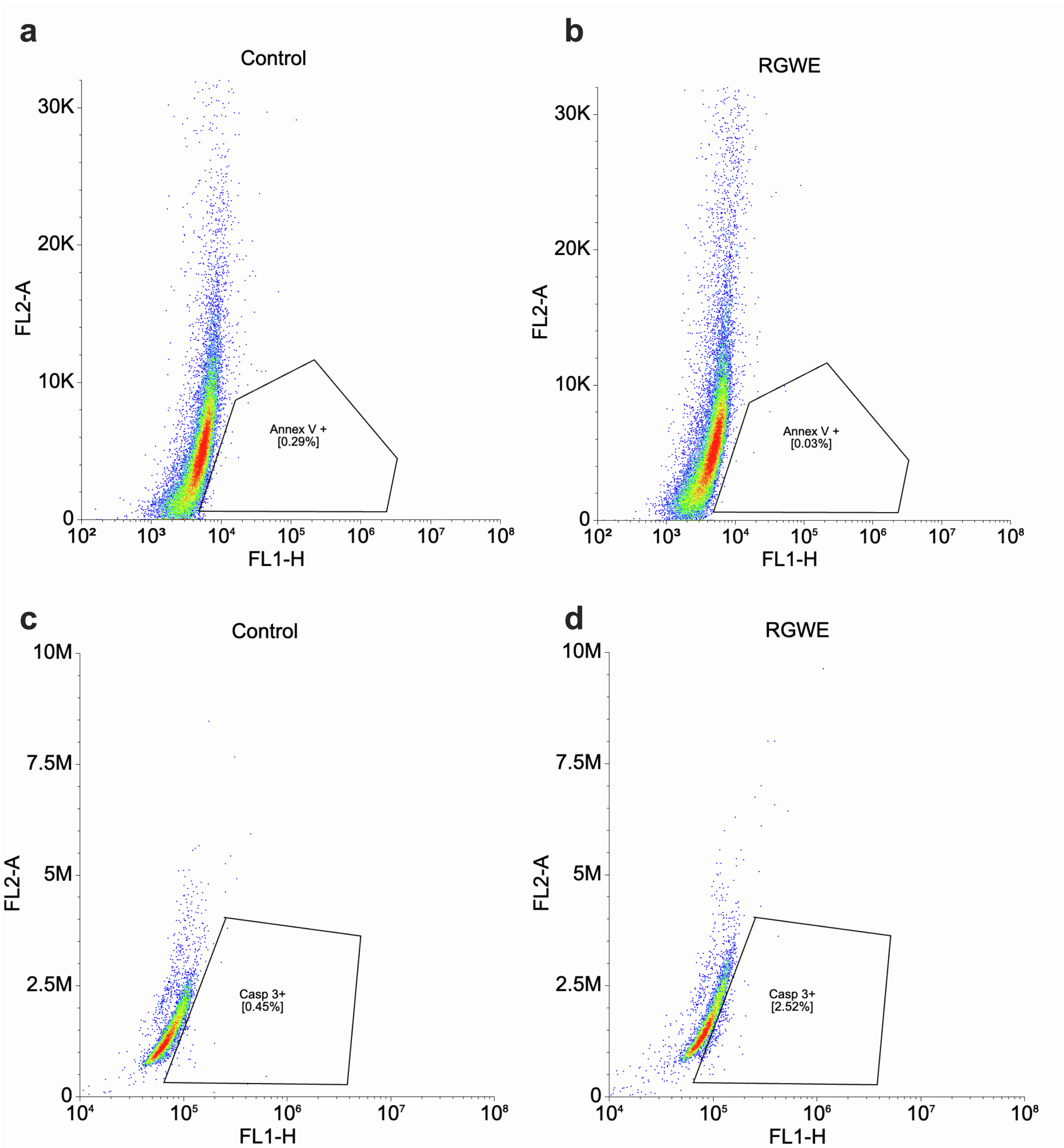


**Figure S1: Apoptosis analysis via a cytofluorometric Annexin V-FITC/PI assay and a caspase-3 activation assay.** U87MG cells were treated with 1 mg/ml RGWE for 48 h. The FL2-A/FL1-H dot plot represents the staining with AnnexV/PI (A-B), and Caspase3- active and PI (C-D) resulted in matching fractions of cells.

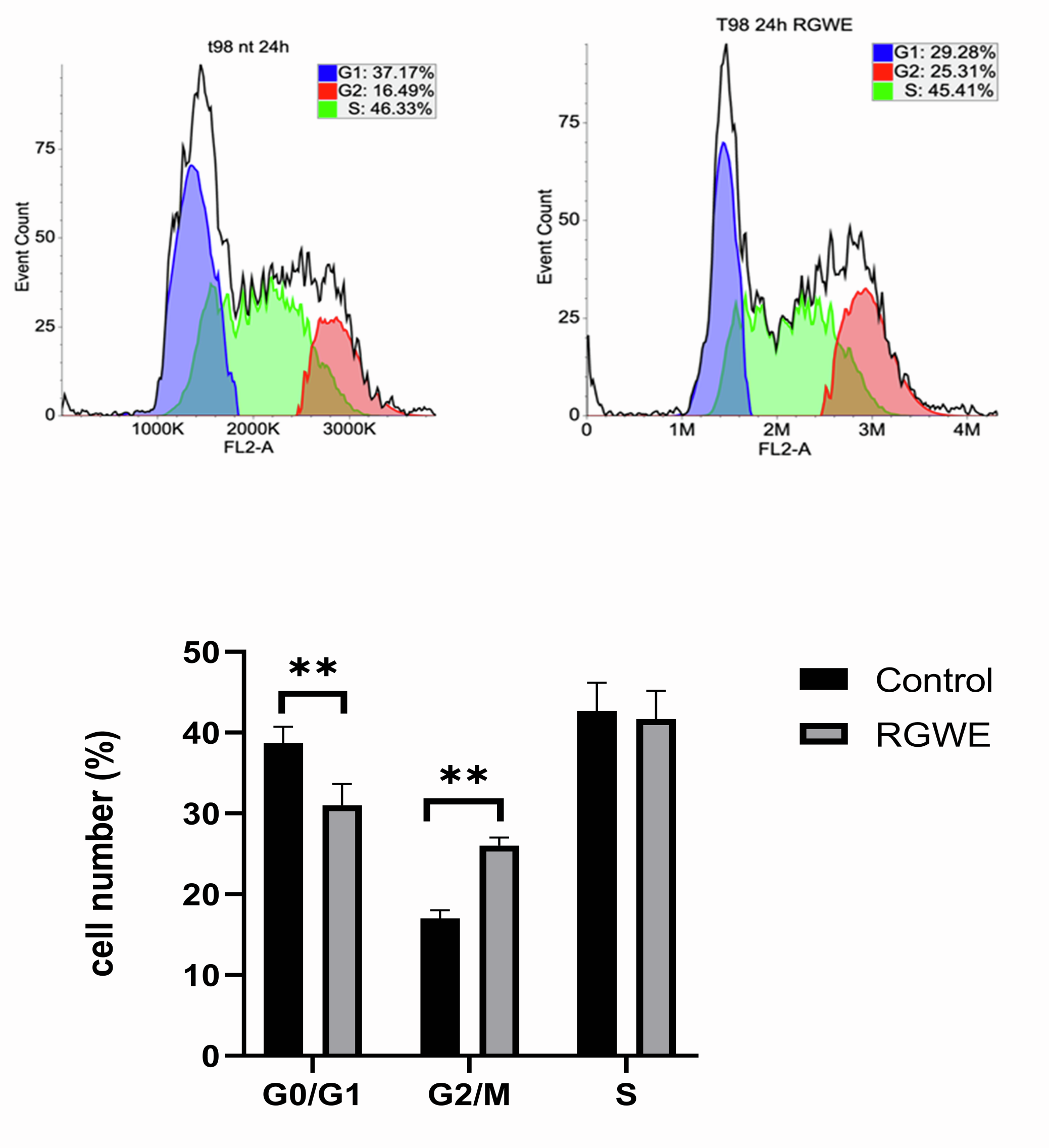


**Figure S2. FACS flow cytometry analysis of the cell cycle in glioblastoma cell lines. (A)** Representative flow cytometry cell cycle analysis of the T98G human glioblastoma cell line treated with 1 mg/ml RGWE for 24 h. The histogram shows the mean ± SD of the percentage of cells accumulated during treatment in each cell cycle phase of three independent experiments. The significance of the data obtained was tested via 2-way ANOVA with Sidak's multiple comparisons (**** $p < 0.0001$, ns: nonsignificant compared with untreated cells, control).

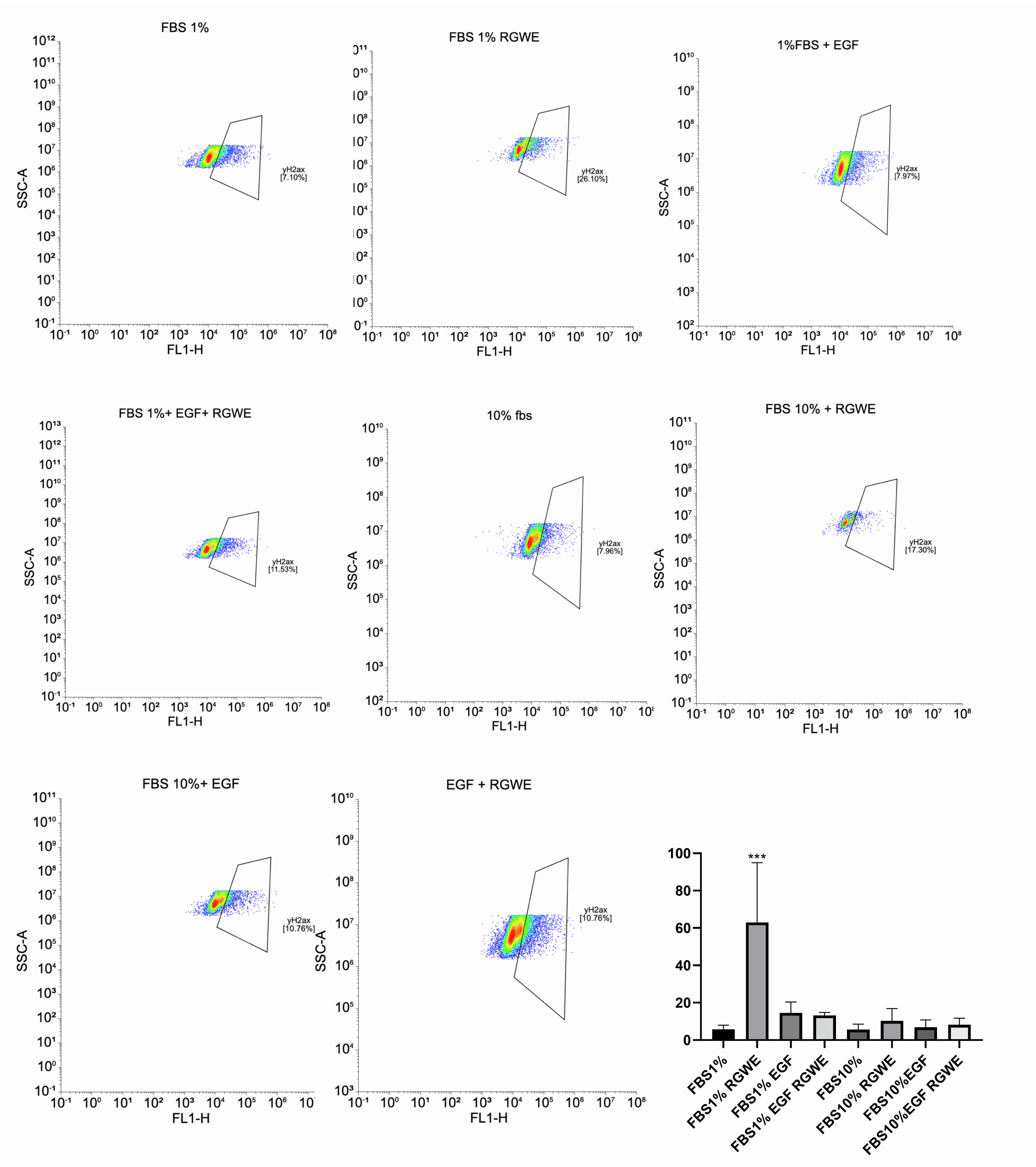


**Figure S3. FACS flow cytometry analysis of γ-H2ax analysis in U-87 MG glioblastoma cell lines. (A)** Representative flow cytometry Dot Plot of U-87 MG cells with 1 mg/ml RGWE for 24 h in the presence or in the absence of FBS1%, FBS 10% and EGF (5nM). The x-axis shows the intensity of fluorescence emitted by γ-H2ax antibody, while the y-axis shows cell size. The gating strategy is shown in the control not tested with the antibody. The histogram shows the mean ± SD of the percentage of cells γ-H2ax positive of three independent experiments. The significance of the data obtained was tested via 2-way ANOVA with Sidak's multiple comparisons (*** $p < 0.001$).

**Table S1: List of DNA oligonucleotides used for real-time quantitative PCR (qPCR). T**he gene identification tag (ID) is shown on the left, the forward primer sequence (FW) and the reverse primer sequence (Rev) is on the right.

| | | |
|---|---|---|
| CCNE1 | Fw | 5' -GTGTCAAGTGGATGGTTCCATTTGCC- 3' |
| | Rv | 5' -GCCCTATTTTGTTCAGACAACATGGC- 3' |
| TP53 | Fw | 5' -CTCAGCATCTTATCCGAGTGGAAGG- 3' |
| | Rv | 5' -CACATGTAGTTGTAGTGGATGGTGG- 3' |
| P14 | Fw | 5' -CTCGTGCTGATGCTACTGAG- 3' |
| | Rv | 5' -ACCAGCGTGTCCAGGAA- 3' |
